# Data Insights for Sustainable Cities: Associations between Google Street View-Derived Urban Greenspace and Google Air View-Derived Pollution Levels

**DOI:** 10.1021/acs.est.3c05000

**Published:** 2023-11-16

**Authors:** Maria E. S. Sabedotti, Anna C. O’Regan, Marguerite M. Nyhan

**Affiliations:** † Discipline of Civil, Structural & Environmental Engineering, School of Engineering & Architecture, University College Cork, Cork T12 K8AF, Ireland; ‡ MaREI, the SFI Research Centre for Energy, Climate & Marine, University College Cork, Ringaskiddy, CorkP43 C573, Ireland; § Environmental Research Institute, University College Cork, Lee Rd, Sundays, Well, Cork T23 XE10, Ireland

**Keywords:** urban greenspace, air pollution, Google Air View, Google Street View, urban analytics, sustainable cities

## Abstract

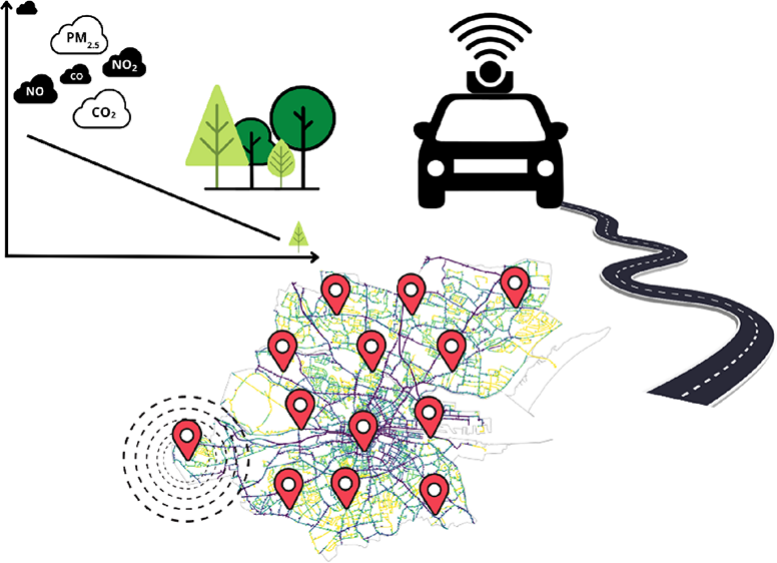

Unprecedented levels of urbanization have escalated urban environmental health issues, including increased air pollution in cities globally. Strategies for mitigating air pollution, including green urban planning, are essential for sustainable and healthy cities. State-of-the-art research investigating urban greenspace and pollution metrics has accelerated through the use of vast digital data sets and new analytical tools. In this study, we examined associations between Google Street View-derived urban greenspace levels and Google Air View-derived air quality, where both have been resolved in extremely high resolution, accuracy, and scale along the entire road network of Dublin City. Particulate matter of size fraction less than 2.5 μm (PM_2.5_), nitrogen dioxide, nitric oxide, carbon monoxide, and carbon dioxide were quantified using 5,030,143 Google Air View measurements, and greenspace was quantified using 403,409 Google Street View images. Significant (*p* < 0.001) negative associations between urban greenspace and pollution were observed. For example, an interquartile range increase in the Green View Index was associated with a 7.4% [95% confidence interval: −13.1%, −1.3%] decrease in NO_2_ at the point location spatial resolution. We provide insights into how large-scale digital data can be harnessed to elucidate urban environmental interactions that will have important planning and policy implications for sustainable future cities.

## Abstract 2

Urban greenspace and pollution levels were quantified in high resolution, accuracy, and scale using Google Air View- and Google Street View-derived methods for a major urban area. Higher levels of greenspace were associated with better air quality.

## 1 Introduction

In the present day, more than 50% of the world’s population lives in cities and this proportion is projected to increase to 68% by the year 2050.^1^ Urbanization and the migration of populations from rural to urban areas have elucidated urban quality-of-life issues as cities are seen as the source and the solution for a range of social, economic, and environmental sustainability problems.^2^ Accelerated rates of urbanization have boosted energy demand and escalated pollution emissions, and this has led to severe air pollution and human exposures to air pollution in urban areas.^3^

According to the World Health Organization (WHO), 99% of our global population breathes unhealthy levels of particulate matter air pollution and lives in places where the WHO’s air quality limits are exceeded.^4^ The primary source of air pollution in cities is vehicular traffic,^5^ but solid fuel combustion from sources including domestic heating and industrial activities also contribute.^6^ Besides emissions, other factors influence air quality in urban environments. These include meteorology, physicochemical transformations, and urban morphology including the size, structure, and growth of cities.^7^ The WHO estimates that 4.2 million premature deaths globally were attributed to ambient air pollution in 2019.^4,8^ Indeed, a plethora of evidence has shown associations between air pollution and adverse health issues including cognitive decline,^9^ chronic respiratory diseases,^10,11^ daily cardiorespiratory hospitalizations,^12^ change in thyroid hormones,^13^ and premature deaths.^14^

Concurrently, the number of studies linking green space, pollution, and health has also increased in recent years, with a growing body of evidence suggesting the human health benefits of urban greenspace.^15,16^ The precise mechanism associating pollution reductions with green urban vegetation is still unclear.^17^ However, potential pathways have been explored, such as the removal of atmospheric pollutants by dry deposition into the surfaces of vegetation;^18−21^ the absorbance of gaseous pollutants through plants stomata;^17,22^ or that fewer pollutant sources exist in extensive greenspace areas.^23^ Nevertheless, research has associated greenspace and decreases in pollution with a lower risk of ischemic heart disease,^24^ better mental health in adolescents,^25^ lower blood pressure in children and teenagers,^26^ diminished respiratory mortality risk among elders,^27^ and benefits to cognitive function.^28^

Considering the evidence linking pollution and greenspace, state-of-the-art research, closely dependent on technological advances,^29^ has been focused on developing new ways of quantifying these variables. Urban air pollution varies greatly over time and short distances because of the irregular spatiotemporal distribution of pollutants sources, and although conventional fixed-site monitoring methods produce extremely high-quality measurements, they often lack the spatial and temporal resolution necessary to characterize this variability.^30,31^ The development of low-cost sensors (LCS) has changed this paradigm and has made it possible to characterize variations in air pollution in urban areas.^32^ The international scientific community has made major strides in calibrating and improving the performance and reliability of LCSs in recent years.^33−35^ Furthermore, drive-by-sensing technology has gained traction as a way of obtaining air pollution data in high spatiotemporal resolution. This has improved our understanding of finely resolved air pollution variations as well as short- and long-term air quality levels in cities.^30,36−39^

In the same way, new technologies have revolutionized the way greenspace metrics are quantified. For example, remote sensing methods, such as satellite imagery, have been applied and have prevailed because of their advantages including large cover areas, synoptic views, and the repeatability of observations.^40−42^ Moreover, the improvement of imagery repositories and tools, such as Google Street View (GSV), have enabled the estimation of vegetation metrics from the street-level perspective.^43^ Therefore, the Green View Index^44^ (GVI) has become a popular way of quantifying street-level greenery because of the availability of high-resolution GSV images and advances in computer vision methods.^40,45−47^ Both remote sensing and ground-based GVI techniques have been used extensively in research linking greenspace to air pollution^41,48−50^ since, from the city perspective, they quantify greenery differently. The GVI captures street greenery, including grass, foliage, bushes, and green walls that could be hidden from an aerial view, while NDVI assesses large green areas that cannot be quantified by the street view.

Studies have investigated the use of mobile-sensing platforms to measure and map urban air pollution^30,36,37,51^ while others have applied GSV to determine greenspace distributions in cities.^40,43,47,52^ Although some studies have analyzed associations between urban greenspace and air pollution,^20,21,41,48−50,53^ none have examined associations between Google Street View-derived urban greenspace levels and Google Air View-measured air quality, where both have been resolved in extremely high resolution along the entire road network of a major city using large-scale digital data. By building on recent advances in high-resolution urban greenspace quantification and air pollution measurements, we addressed this knowledge gap by investigating the spatial associations between novel greenspace and air pollution metrics determined using large-scale digital data on the entire road network of Dublin City, Ireland, while controlling for important factors. It was intended that the results of this study would have important implications for research and policy for smart and sustainable future cities.

## 2 Methods

### 2.1 Study Protocol and Study Domain

This study examined associations between street-level urban greenspace and air quality in Dublin City. Dublin is a low-rise^54^ large urban area located in the east of Ireland, which has a population of 588,233 people^55^ and an area of 118 km^2^.^56^ 35% of Dublin City is covered by greenspace infrastructure^57^ including approximately 300,000 trees. 19.3% of these trees are located close to roads.^58^ Regarding air pollution, according to the European Environmental Agency,^59^ Dublin was the 25th cleanest city in Europe between the years 2021 and 2022, with a mean annual fine particulate matter level of 7.4 μg/m^3^.

In this study, air pollution data in high spatial resolution was acquired from Google, Aclima, and Dublin City Council through the Project Air View Dublin monitoring campaign, which equipped a Google Street View Car with an air pollution sensing platform.^60,61^ The monitoring campaign collected 5,030,143 pollution measurements throughout Dublin city on 24,694 road segments. Street-level urban greenspace was quantified by using two methods. First, it was determined along the entire road network of Dublin using 403,509 Google Street View images collected at 67,265 point locations. Second, it was assessed using satellite imagery, also along the road network. Parks and green areas that were not immediately located along the streets were masked out. Computer vision image segmentation methods were applied to Google Street View images in order to determine street-level urban greenspace, while satellite imagery and computational algorithms were used to estimate overhead aerial view urban greenspace coverage.

Greenspace analyses were conducted at all point locations and also within buffer zones. Each point corresponded to the midpoint of each 50 m road segment on which air pollution measurements were made. For the buffer zone analysis, buffers were taken around each point location. The point locations accounted for the finest spatial resolution studied, whereas buffer zones enabled a better understanding of the impact of larger urban greenspace zones on air pollution through the inclusion of spatial averaging effects, while also supporting a comparison of the results to published literature.^49^ The data sets that were processed and analyzed and the statistical analyses used to understand associations between urban greenspace and air quality in high spatial resolution are described in the following sections.

### 2.2 Google Air View Air Pollution Data

Google Street View cars are custom-modified vehicles equipped with roof-mounted camera systems that are used to collect imagery on road networks globally.^62^ For the Air View Project, Google partnered with Aclima to custom-modify and equip these Street View vehicles with specialized instruments for precisely measuring pollution concentrations. In Dublin, the first electric Google Street View car was equipped with Aclima’s mobile air measurement and analysis platform, and this was used to quantify and map air pollution parameters and greenhouse gas emissions.^60^ The pollutants measured were particulate matter of size fraction less than 2.5 μm (PM_2.5_) (including size-resolved particle counts from 0.3 to 2.5 μm), nitrogen dioxide (NO_2_), nitric oxide (NO), carbon monoxide (CO), and carbon dioxide (CO_2_).^63^

The Google Street View car collected geolocated concentrations of air pollution and CO_2_ every second (1 Hz), street by street, while driving with the flow of traffic at normal speeds. The 5,030,143 data points collected were processed over 24,694 road segments of length 50 m. All road segments were visited at least six times during the study period, where each visit was defined as driving on the road segment at least once in a 4 h time frame. The median number of visits to each road segment was 14. All measurements were processed and statistically analyzed by Aclima using methodologies described in Apte et al.^30^ and Miller et al.,^51^ in order to generate the estimates of air pollution (PM_2.5_, NO_2_, NO, CO, and CO_2_) for all 24,694 road segments. Data collection for Dublin City took place from May 2021 until August 2022, from Monday to Friday between 9 am and 5 pm, and thus represented typical daytime, weekday air quality.

### 2.3 Urban Greenspace Metrics

#### 2.3.1 Google Street View-Derived Urban Street-Level Green View Index

The GVI is a method of assessing vegetation at street level.^43^ Also called eye-level greenery, it was first proposed by Aoki in 1987^44^ and has gained traction in recent years with the emergence and advancement of imagery tools including Google Street View (GSV), which supply images in high spatial resolution.^49^ Street-level urban greenspace was quantified at 67,265 Green Point locations in Dublin City, using 403,509 GSV images. These points were generated every 50 m along the city’s road network. For each point, six GSV images were downloaded to obtain a viewing angle at 60° intervals. Using computer vision methods outlined in O’Regan et al.,^47^ the GVI was calculated for each point using the following equation:
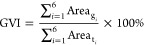
1where Area_g*i*_ is the number of green pixels in image *i* and Area_t*i*_ is the total number of pixels in image *i*. The values of GVI range between 0 and 100, with larger values representing a greater density of visual greenspace. In this study, GVI values were attributed to the closest point locations, while for buffer zones, the mean of all GVI points within each zone was computed.

#### 2.3.2 Satellite Imagery-Derived Normalized Difference Vegetation Index

The Normalized Difference Vegetation Index (NDVI) is a greenspace metric that is computed through the use of remote sensing images and therefore assesses aerial view greenspace.^43,52^ The NDVI metric was used for comparison with the high-resolution urban greenspace metric (GVI). The NDVI for Dublin City was determined using Sentinel-2 satellite imagery data in 10 m × 10 m grid-cell resolution.^64^ Aggregated satellite imagery for March 2022 was used. The data exhibited a low percentage of clouds (less than 5%), and thus the imagery was of higher quality relative to imagery from other times during the year. Before computing the NDVI, an Fmask filter was employed in order to detect clouds and cloud shadows in the Landsat imagery, using methods recommended by Zhu and Woodcock.^65^ The filtered pixels were removed before further analysis. The NDVI was determined as follows:
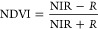
2where NIR represents the near-infrared band (Band 8) and *R* represents the red band (Band 4) in the Sentinel-2 imagery. NDVI values range from −1 to +1, with higher values indicating more green vegetation. The NDVI values were extracted at the exact location of each GVI point. This masked out parks and green areas that were not immediately located along streets. This resulted in a total of 67,265 NDVI points. For the buffer zone analysis, the mean of all NDVI points within each buffer zone was computed, whereas for the point locations, NDVI values were assigned to the closest point.

#### 2.3.3 GVI and NDVI Ratio

A third urban greenspace parameter was also considered, represented by the ratio between GVI and NDVI. This additional metric was proposed by Larkin and Hystad^66^ and later adopted by O’Regan et al.^49^ in an effort to obtain more information on the street-view greenspace setting since it can capture unique information present in both greenspace metrics (GVI and NDVI). For this, the values of GVI and NDVI were normalized using the following equations, and the ratio (GVI_norm_/NDVI_norm_) ranged from 0 to 1.
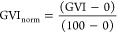
3
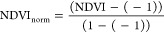
4

### 2.4 Meteorology and Traffic Data

Precipitation and temperature data were obtained from Met Éireann.^67^ The 1 km × 1 km gridded data set contains monthly values of precipitation and temperature for the study period. Averages for the full study duration were attributed to each pollution point location. Traffic counts were provided by IDASO^68^ and Transport Infrastructure Ireland’s^69^ fixed traffic counters for the study period. Only counts on weekdays during daytime work hours were used to establish the average hourly traffic volumes, following the same collection pattern used by Google and Aclima.^60^ Traffic counts were provided for different vehicle types, and these were converted to passenger car units (PCUs). Total PCUs were used for the analysis. Population density was determined using census statistics.^70^ The values were obtained at the Small Area level (the smallest administrative unit), and it was assumed that the population was uniformly distributed within these. The population density was attributed to each pollution point, consistent with their location.

### 2.5 Statistical Analysis

#### 2.5.1 Descriptive Statistics

After collecting and processing the data, the *z*-score, Mahalanobis distance, skewness, and kurtosis tests were applied in order to discard outliers. Descriptive statistics for all of the greenspace and pollution metrics were computed. The approach used for modeling associations between urban greenspace and air pollution is described in the following subsections.

#### 2.5.2 Testing for Spatial Autocorrelation

The Moran’s *I* test was first computed for each pollutant variable, in order to evaluate the potential of spatial autocorrelation in the data, as indicated by Hao and Liu.^71^ It was determined using the following equation:
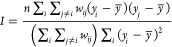
5where *y* is the variable of interest and *w*_*ij*_ is the weight assigned to the spatial weight matrix for locations *i* and *j*. The values vary from −1 to 1. Values higher than 0.3 indicated a positive spatial autocorrelation, while values lower than −0.3 indicated a negative spatial autocorrelation.

The weight matrix used for the Moran’s *I* test and subsequent analyses was a kernel weight matrix with adaptive bandwidths and a triangular function. This method is based on the distance between observations and assumes that spatial similarity decreases with separation. The matrix was row standardized.

#### 2.5.3 Spatial Regression

Since all the pollutants (PM_2.5_, NO_2_, NO, CO, and CO_2_) indicated positive spatial autocorrelation using the Moran’s *I* test (see Table S1 in the Supporting Information), a spatial autoregressive model (SAR) was used as has been applied in previous research.^71−74^ Lagrange multiplier and robust LM tests were run to identify the best SAR model to use in the analysis. Considering that both tests presented significant results for almost all pollutants in all analyses (see Tables S2, S3, S4, S5 and S6 in the Supporting Information), both the spatial lag model (SLM) and the spatial error model (SEM) were applied and their results compared. The SLM was chosen as the best-fit model for our data considering Moran’s *I* test for their residuals.

The SLM addresses spatial autocorrelation in dependent variables by considering the effect of neighboring measures.^73^ The SLM was applied to model associations between urban greenspace and air pollution whereby GVI, NDVI, and GVI:NDVI were evaluated as the independent variables and PM_2.5_, NO_2_, NO, CO, and CO_2_ as the dependent ones. Meteorology, traffic, and population data were included as control variables in the models, similar to previous studies.^1,49^ The SLM followed the general form:
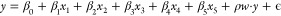
6where *y* refers to the level of pollution (PM_2.5_, NO_2_, NO, CO, and CO_2_), β_0_ is the intercept of the regression line, *x*_1_ refers to the level of greenspace (GVI, NDVI, and GVI:NDVI), *x*_2_ is the temperature, *x*_3_ is the level of precipitation, *x*_4_ is the population density, *x*_5_ is the traffic counts, β_1,2,3,4,5_ refers to the estimated direct coefficients for each of the predictor variables, ρ is the spatial autoregressive coefficient, *w* symbolizes the spatial weight matrix, and ϵ is the error term.

Subsequently, we computed the spatial spillover effect of the model, using the following equation:
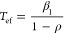
7where *T*_ef_ represents the total effect of the greenspace metric on the pollutant, β_1_ is the estimated direct coefficient for the greenspace metric, and ρ indicates the spatial autoregressive coefficient.

Three different models were run for each pollution dependent variable, analyzing the effects of GVI, NDVI, and GVI:NDVI separately on each pollution variable for all point locations and for all buffer zones studied. *P*-values less than 0.05 were considered statistically significant, and values less than 0.01 were highly statistically significant. In this study, the associations were reported as percentage difference in pollution per interquartile range (IQR)^75^ increase in greenspace levels. Data processing and analyses were completed using QGIS version 3.28.3,^76^ R version 4.2.3,^77^ and Python version 3.11.3.^78^

## 3 Results

### 3.1 Air Pollution and Urban Greenspace

Summary statistics (mean, standard deviation, minimum, and maximum) were computed for urban greenspace (GVI, NDVI, and GVI:NDVI) metrics and for pollution parameters (PM_2.5_, NO_2_, NO, CO, and CO_2_) for all point locations and all buffer zones of radii 100, 300, 500, 1000, and 2000 m (see Table 1). The IQR was computed for GVI, NDVI, and GVI:NDVI for the point locations and all of the buffer zones studied (see Table 2 for details).

**Table 1:** Summary Statistics Including the Mean (μ), Standard Deviation (δ), Minimum (min), and Maximum (max) for Greenspace Metrics (GVI, NDVI, and GVI:NDVI) and Pollution Parameters (PM_2.5_, NO_2_, NO, CO, and CO_2_), for All Point Locations and All Buffer Zones (*n* = 24,694)^a^

		**Buffer zone radius**				
	**Points**	100 m	300 m	500 m	1000 m	**2000 m**
	μ (δ) min, max					
**GVI (%)**	14.15 (9.94)	14.27 (7.59)	14.61 (6.00)	14.62 (6.01)	14.85 (4.62)	14.86 (3.36)
	0.00, 51.85	0.00, 43.57	2.93, 35.56	2.94, 35.56	6.00, 28.29	8.64, 23.16
**NDVI**	0.12 (0.07)	0.12 (0.06)	012 (0.05)	0.12 (0.05)	0.13 (0.04)	0.13 (0.04)
	–0.03, 0.39	–0.01, 0.31	0.01, 0.25	0.01, 0.25	0.03, 0.22	0.06, 0.20
**GVI:NDVI**	0.25 (0.17)	0.25 (0.13)	0.25 (0.10)	0.25 (0.10)	0.26 (0.07)	0.26 (0.05)
	0.00, 0.87	0.00, 0.74	0.06, 0.60	0.06, 0.60	0.12, 0.47	0.16, 0.39
**PM**_**2.5**_**(μg/m**^**3**^**)**	6.49 (2.00)	6.48 (2.00)	6.48 (2.01)	6.48 (2.01)	6.51 (2.03)	6.54 (2.06)
	2.02, 18.74	1.96, 18.74	1.97, 18.74	1.97, 18.74	1.96, 18.74	1.96, 19.33
**NO**_**2**_**(μg/m^3^)**	17.62 (15.45)	17.75 (15.97)	17.93 (16.35)	17.93 (16.35)	18.23 (17.96)	18.38 (18.07)
	0.38, 252.80	0.38, 280.57	0.38, 233.50	0.38, 233.50	0.38, 286.02	0.38, 286.02
**NO (μg/m**^**3**^**)**	24.56 (42.16)	25.39 (45.09)	26.29 (51.19)	26.29 (51.17)	26.77 (53.81)	26.99 (54.04)
	0.16, 735.13	0.15, 738.90	0.15, 860.35	0.15, 860.35	0.15, 871.16	0.15, 871.16
**CO** (mg/m^3^)	860.90 (21.30)	808.90 (21.46)	809.00 (21.37)	809.00 (21.36)	809.00 (21.41)	809.10 (21.51)
	740.20, 895.30	746.50, 895.80	746.50, 892.30	746.50, 892.30	746.50, 891.20	740.20, 896.70
**CO**_**2**_(mg/m^3^)	0.37 (0.05)	0.37 (0.05)	0.37 (0.05)	0.37 (0.05)	0.37 (0.05)	0.37 (0.05)
	0.25, 0.58	0.25, 0.58	0.25, 0.58	0.25, 0.58	0.25, 0.58	0.25, 0.58

a GVI values range from 0 to 100 (%); NDVI values vary between −1 and 1; and GVI:NDVI ratios vary from 0 to 1. For all of the metrics, higher values correspond with a greater level of greenspace.

**Table 2:** Interquartile Ranges (IQR) for Urban Greenspace Metrics (GVI, NDVI, and GVI:NDVI) for All Point Locations and for All Buffer Zones (*n* = 24,694) of Varying Radii within Our Study Domain

		GVI (%)	**NDVI**	**GVI:NDVI**
**Buffer zone radius**	Points	14.370	0.108	0.243
	100 m	11.136	0.091	0.183
	300 m	9.137	0.074	0.150
	500 m	9.139	0.074	0.150
	1000 m	7.048	0.070	0.114
	2000 m	5.503	0.066	0.087

**Figure 1:**
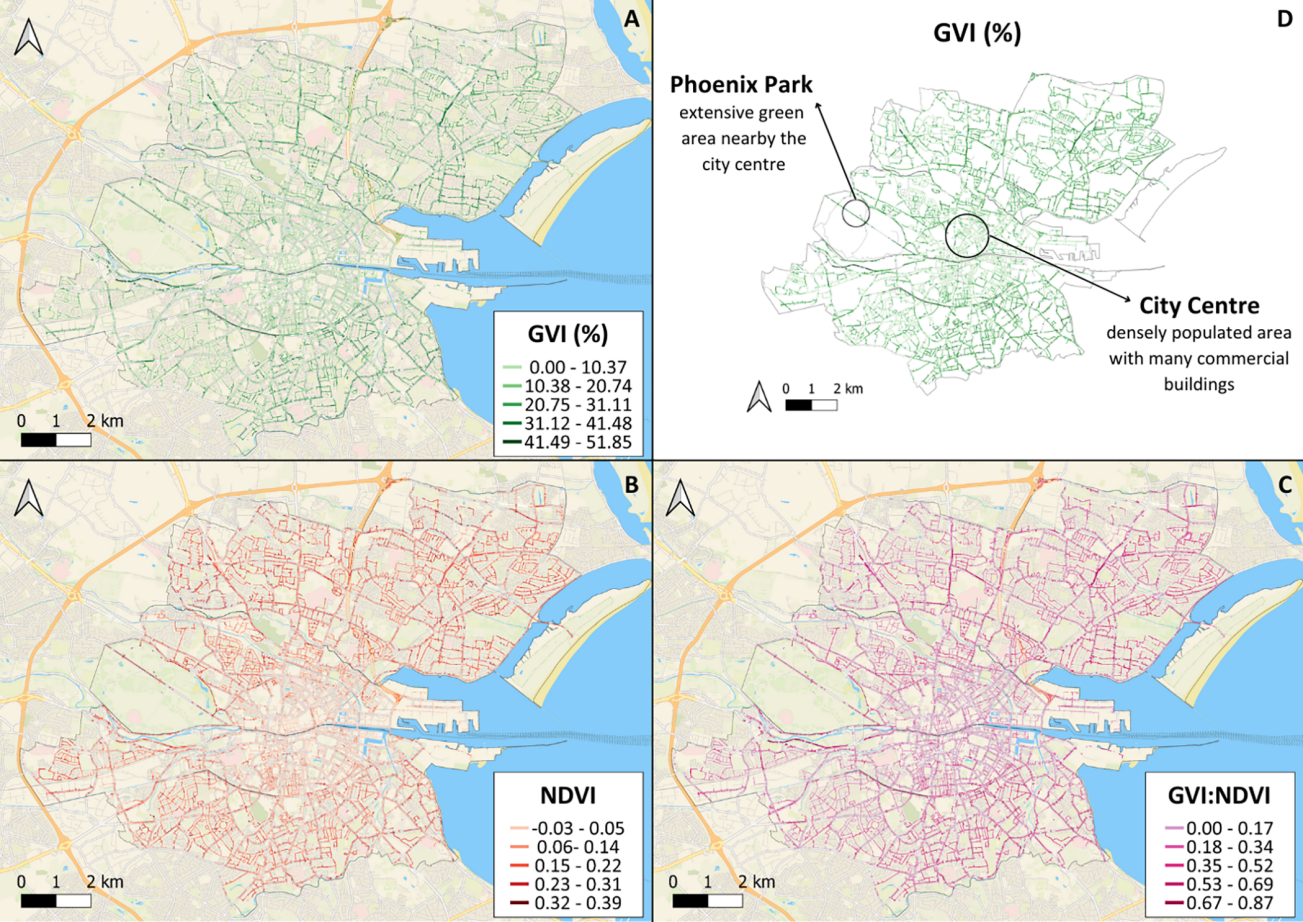
Map of urban greenspace metrics (GVI, NDVI, and GVI:NDVI) computed in high spatial resolution (67,265 point locations) on the entire road network of Dublin City (A, B, C, respectively). The GVI map (D) highlights contrasting sites representing a dense urban City Center area and a major urban park (Phoenix Park), which assists in the visual interpretation of the urban greenspace metrics computed across the city. See Supporting Information for larger detailed urban greenspace maps (Figure S1, S2, S3 and S4).

Figure 1 displays the distribution of urban greenspace metrics (GVI, NDVI, and GVI:NDVI) across the road network of Dublin City. From a visual analysis of the maps, it was observed that the city center area had a modest quantity of greenspace along its road network relative to other areas, for all three metrics. The city center area is densely populated with many commercial buildings (Figure 1). It is also possible to distinguish places where all urban greenspace metrics have relatively higher values on roads within extensive green areas and parks, for example, Phoenix Park (Figure 1).

**Figure 2:**
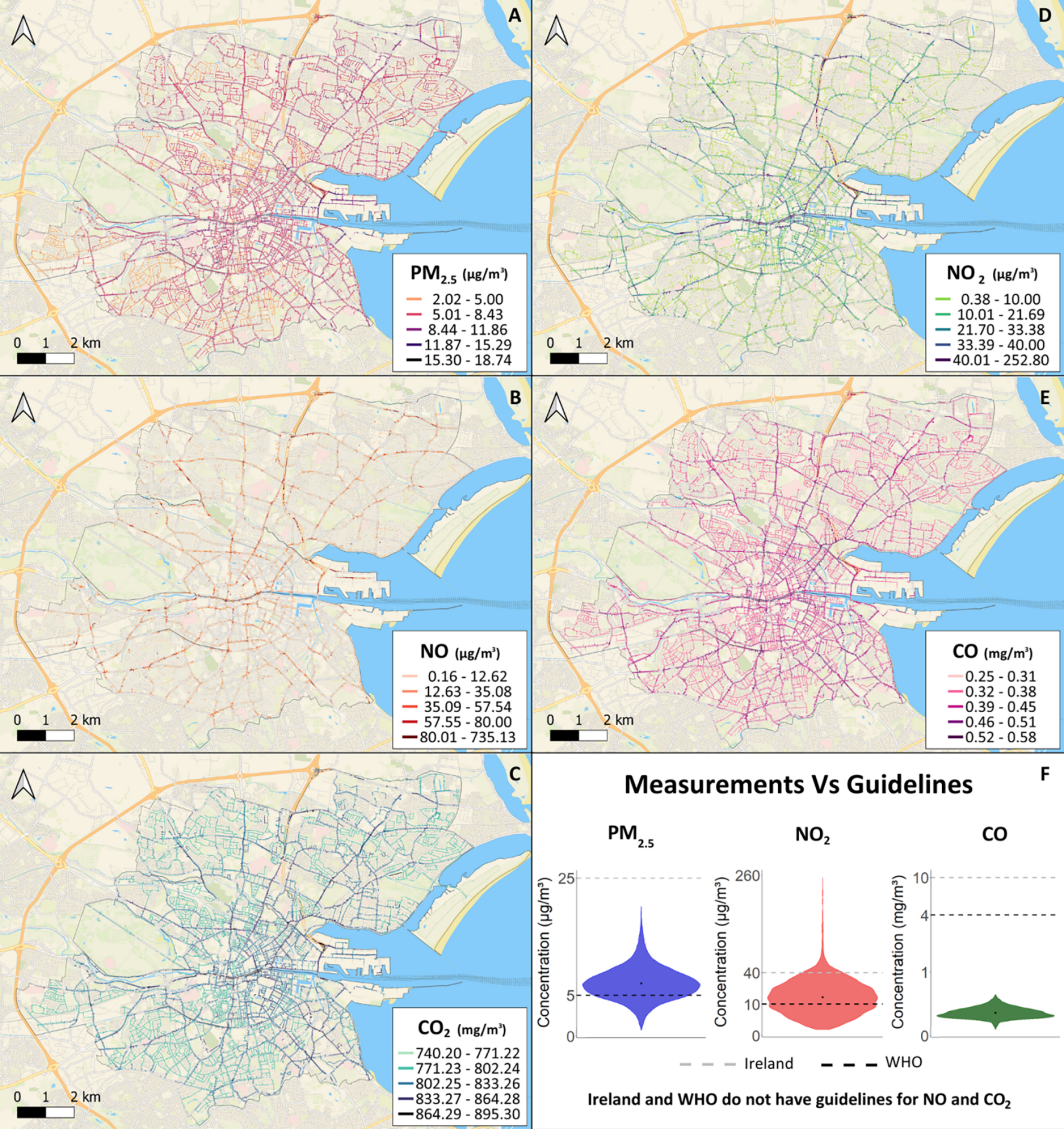
Maps of Google Air View pollution parameters (PM_2.5_, NO_2_, NO, CO, and CO_2_) determined in high spatial resolution and computed using 5,030,143 point measurements on 24,694 road sections (50 m road segments) across the entire road network of Dublin City (A, B, C, D, E). We also show graphs depicting comparisons of citywide pollution distributions with national Irish and international World Health Organization limits (F). The mean PM_2.5_ and NO_2_ values lie above the WHO recommendations. In contrast, all of the values measured for CO are below the guideline limits. No guideline limits are available for CO_2_ and NO. Please see the Supporting Information for larger detailed maps of pollution parameters (Figures S5, S6, S7, S8, S9 and S10).

We also spatially mapped and compared pollution (PM_2.5_, NO_2_, NO, CO, and CO_2_) distributions across the entire road network of Dublin City, as shown in Figure 2. The city center region and major arterial roadways emanating from and concentrically situated around the urban center exhibited higher concentrations of all pollution parameters relative to other areas. Although NO levels were elevated along major roadways, the concentrations were not necessarily higher in the urban center relative to other areas. We also compared the measurements of PM_2.5_, NO_2_, and CO with the current Irish Environmental Protection Agency (EPA) and WHO guidelines (Figure 2F).^79,80^ The mean PM_2.5_ and NO_2_ levels were higher than the WHO guidelines and lower than the EPA guidelines. Some air pollution measurements for NO_2_ were substantially higher than the limits proposed to protect human health. In contrast, all of the measured values of CO were lower than both guidelines. The WHO and EPA have no guidelines set for NO and CO_2_.

### 3.2 Associations between Urban Greenspace and Pollution

**Figure 3:**
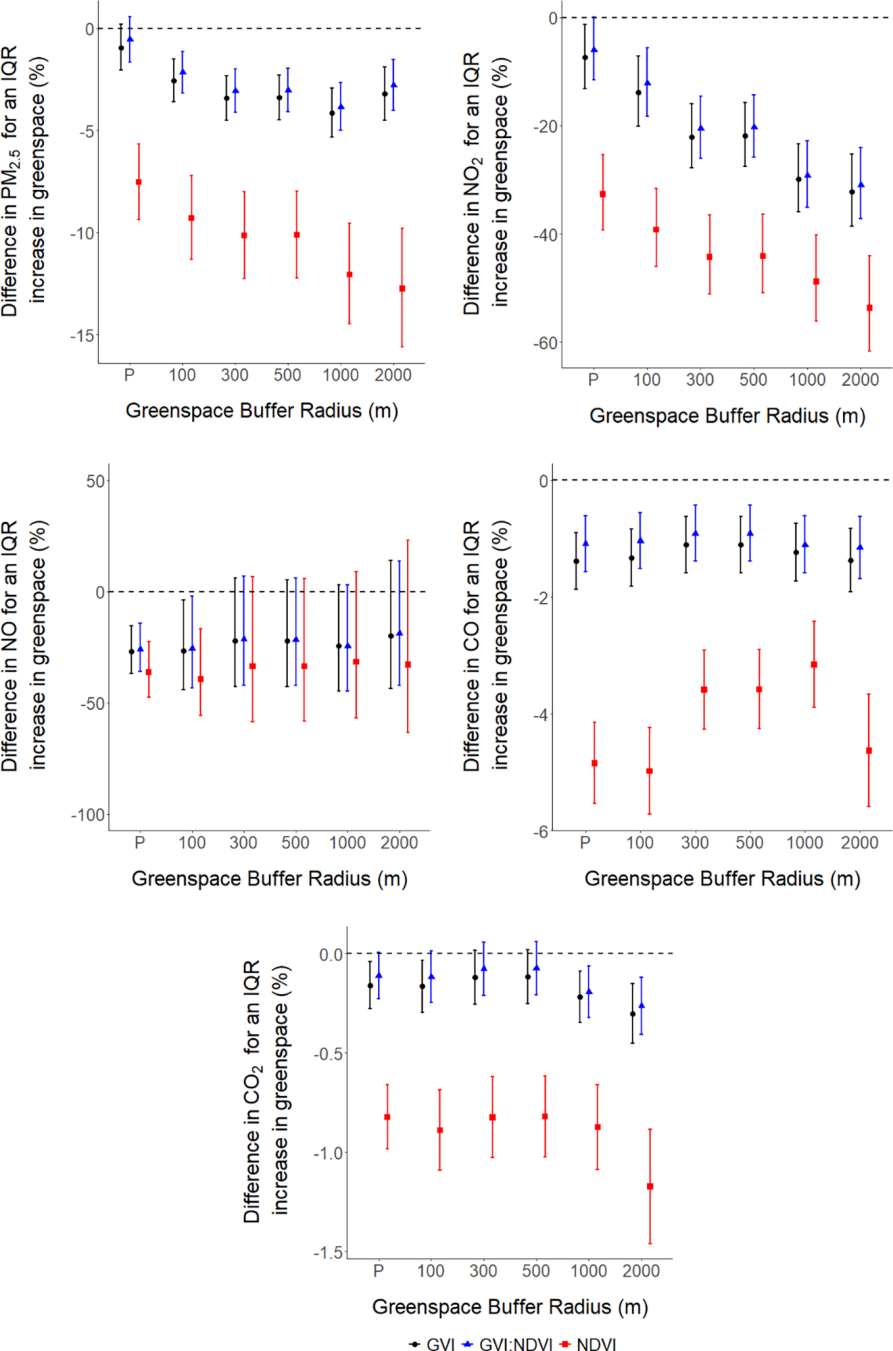
Associations between urban greenspace (GVI, NDVI, and GVI:NDVI) and pollution parameters (PM_2.5_, NO_2_, NO, CO, and CO_2_) determined for point locations (P) and buffer zones of radius 100, 300, 500, 1000, and 2000 m (*n* = 24,694). All models were adjusted for traffic, average daily precipitation, temperature, and relative humidity. The *y*-axis represents the difference in percentage for PM_2.5_, NO_2_, NO, CO, and CO_2_ for an IQR increase in urban greenspace (GVI, NDVI, and GVI:NDVI). The error bars represent the 95% confidence intervals. Please note that the *y*-axis scales vary across the graphs.

The results of the regression analysis consistently indicated highly statistically significant *(p <* 0.001) associations between higher levels of urban greenspace (GVI, NDVI, and GVI:NDVI) and decreased pollution levels (PM_2.5_, NO_2_, NO, CO, and CO_2_) for almost all scenarios studied (point locations and buffer zones of radii 100, 300, 500, 100, and 2000 m) with a small few exceptions (see Figure 3 and Table S8 for details).

Considering the highest spatial resolution studied (point locations), all inverse associations between urban greenspace and air pollution were statistically significant (*p* < 0.05) except for PM_2.5_. An IQR increase in GVI was significantly (*p* < 0.001) associated with decreases in NO_2_ [−7.39% (95% confidence interval (CI): −13.12, −1.28)] for the point locations, while the buffer zones presented larger negative associations. An IQR increase in GVI was associated with declines in CO [−1.39% (95% CI: −1.87, −0.90)] and NO [−26.85% (95% CI: −36.87, −15.25)], and these associations tended to be slightly stronger than associations observed for these same pairs of variables for the buffer zones. Furthermore, an IQR increase in GVI was associated with a decrease in CO_2_ [−0.16% (95% CI: −0.28, −0.05)].

Comparing the greenspace metrics at the point location resolution, NDVI consistently presented larger inverse associations with all air pollution variables than GVI and GVI:NDVI. For example, an IQR increase in NDVI was associated with a 32.69% decrease [95% CI: −39.28, −25.39] in NO_2_, while IQR increases in GVI and GVI:NDVI were associated with a 7.39% decrease [95% CI: −13.12, −1.28] and a 5.95% decrease [95% CI: −11.58, −0.042] in NO_2_, respectively.

Higher levels of urban greenspace were significantly (*p* < 0.001) associated with lower levels of PM_2.5_ air pollution across all greenspace metrics and buffer zones, except for both GVI and GVI:NDVI metrics for the point location resolution. For instance, an IQR increase in GVI was associated with a 4.12% decrease [95% CI: −5.32, −2.92] in PM_2.5_ for the 1000 m-radius buffer zone. The associations between greenspace and PM_2.5_ were slightly attenuated for the GVI:NDVI ratio relative to the GVI metric. The magnitude of associations was larger for the NDVI metric relative to both GVI and GVI:NDVI. For example, for the 2000 m radius buffer zone, an IQR increase in NDVI was associated with a 12.74% decrease [95% CI: −15.59, −9.80] in PM_2.5_.

Similar results were observed for NO_2_. Higher levels of urban greenspace were significantly (*p* < 0.001) associated with lower levels of NO_2_ air pollution across all buffer zones, with the exception of the GVI:NDVI metric for the point locations resolution. For instance, an IQR increase in GVI was associated with a 32.24% decrease [95% CI: −38.62, −25.20] in NO_2_ for the 2000 m-radius buffer zone. These associations were slightly attenuated for the GVI:NDVI metric, while for NDVI, decreases in NO_2_ observed were larger relative to GVI and GVI:NDVI. An IQR increase in NDVI was associated with a 53.70% decrease [95% CI: −61.69, −44.05] in NO_2_ for the 2000 m-radius buffer zone.

Only the point locations and the 100 m buffer resulted in significant (*p* < 0.05) inverse associations between all greenspace metrics and NO. The largest decrease in NO [−39.37% (95% CI: −55.76, −16.90] was observed for an IQR increase in NDVI for the 100 m-radius buffer zone. In addition, IQR increases in urban greenspace were associated with decreases in CO levels. Of the metrics and areas studied, the strongest negative association observed for CO [−4.98% (95% CI: −5.72, −4.24)] was for an IQR increase in NDVI for the 100 m-radius buffer zone. Furthermore, IQR increases in GVI and GVI:NDVI were associated with decreases in the CO_2_ levels for all point locations and buffer zones. An IQR increase in GVI was associated with a 0.30% decrease [95% CI: −0.45, −0.15] in CO_2_ for the 2000 m-radius buffer zone.

The magnitude of the associations between higher urban greenspace levels and decreased pollution levels tended to increase as the buffer zone increased for PM_2.5_ and NO_2_. This implies that larger zones and areas may warrant further investigation.

## 4 Discussion

The importance of greenspace in mitigating air pollution and its associated impact on human health has been shown in several recent studies, with some authors highlighting its impact on improved cognitive performance,^81^ improved lung function,^42^ lower chronic health conditions including hypertension and diabetes,^82^ decreased hospitalizations from cardiovascular diseases,^83^ and decreases in mortality.^84^ In order to better understand interactions between urban greenspace and air pollution, we investigated associations between urban greenspace levels (determined using both GSV- and satellite imagery-derived metrics) and Google Air View-derived air quality measurements resolved in extremely high spatial resolution along the entire road network of a major city. A spatial lag regression model was employed to understand the relationship between urban greenspace and pollution metrics on varying spatial scales.

The findings demonstrated significant (*p* < 0.001) associations between higher levels of urban greenspace (GVI, GVI:NDVI, and NDVI) and decreased pollution (PM_2.5_, NO_2_, NO, CO, and CO_2_) levels for almost all scenarios studied. For example, an IQR increase in GVI was associated with a 4.12% [95% CI: −5.32, −2.92] decrease in PM_2.5_ for the 1000 m-radius buffer zone studied. Higher levels of GVI:NDVI and NDVI were also significantly (*p* < 0.001) associated with decreased PM_2.5_. Similar results were observed by Irga et al.,^85^ who reported that sites with lower urban greenspace densities exhibited higher concentrations of PM_2.5_ in Sydney, Australia. Ai et al.^41^ explored the interaction between vegetation and air pollution and concluded that a 0.1-unit change in NDVI was associated with a 1.9 μg/m^3^ reduction in PM_2.5_, which was analogous with our findings. The inverse association between greenspace and PM_2.5_ that we observed was also observed in Chen et al.^48^ who reported that urban areas with higher urban greenspace coverage consistently exhibited lower PM_2.5_ concentrations across five megacities in China.

Our research findings and the direction of association between urban greenspace and other pollution parameters were consistent with those of existing studies. In our study, for an IQR increase in GVI, we observed decreases of 7.39% [95% CI: −13.12, −1.28] in NO_2_, 0.15% [95% CI: −0.28, −0.04] in CO_2_, 26.85% [95% CI: −36.87, −15.25] in NO, and 1.39% [95% CI: −1.87, −0.90] in CO for the point location analyses. Anderson and Gough^53^ reported that greenspace infrastructure in Ontario, Canada, was associated with an average reduction of 65% for NO_2_ and 6% for CO_2_, regardless of the urban morphology. Furthermore, Klinberg et al.^86^ examined the urban landscape in Gothenburg City in Sweden and observed that green vegetation near busy traffic roads was associated with lower NO_2_ concentrations.

Some minor discrepancies were observed between our results and other findings in relation to the buffer zones studied. Xu et al.^50^ explored the impact of street greenery on street-level PM_2.5_ concentrations using street-view imagery from a three-dimensional perspective. In this study, the 250 m-radius buffer zone (from a range between 50 and 500 m) resulted in the strongest negative correlation between greenspace and air pollution. This was slightly different from our research, which found that the larger the radius of the GVI buffer zone, the greater the decrease in PM_2.5_ pollution. For larger buffer zones, more greenspace is captured relative to the highest spatial resolutions (e.g., point locations and smaller buffers) and these greenspace metrics for large buffer zones tend to show a stronger association with pollution metrics, which were kept the same for all spatial areas studied. Among their findings, Xu et al.^50^ also highlighted the importance of the GVI in terms of characterizing street greenery and complementing the two-dimensional perspective or the NDVI parameter. In our study, the spatial lag regressions considering NDVI exhibited the largest associations. Xu et al.^50^ used a geographically weighted regression model within which the effects of GVI and NDVI were summed in the analyses, while we normalized our GVI and NDVI metrics and computed their ratio before applying the spatial lag regression, which may explain the difference in the findings.

The major strength and novelty of this study lies in the fact that both pollution and greenspace metrics were resolved in extremely high spatial resolution along the entire road network of an entire city using large-scale digital data. First, the Google Project Air View data represent expected weekday daytime street-level concentrations of pollutants where points have been resolved approximately 50 m from each other. The drive-by sensing methodology employed ensured that a dense spatiotemporal data set was collected,^39^ which would not be achieved even for a dense distributed network of air quality sensors.^37^ Furthermore, it enabled the visualization of pollutant hotspots across the city. This is a crucial characteristic since local variations in air pollution can impact immensely on public health.^30^ Second, the use of GSV imagery to quantify street-level greenspace, also collected every 50 m on the road network, provided a detailed map in high spatial resolution.^49^ For analyses of the associations between urban greenspace and air pollution, the dependent and independent variables were both resolved in unprecedented detail. This directly improved the rigor of the statistical analysis results since sample size directly impacts on regression suitability and statistical power.^87^ It is also important to highlight that this study does not imply causation between increased greenspace and decreased air pollution. Rather, we investigated the association between the variables in different spatial resolutions while controlling for important factors.

Regarding reproducibility, this study is scalable globally. This is as a result of technology advances including fast response environmental sensing platforms^30,36,37,39,60^ and large-scale visual imagery data sets combined with computer vision methods,^40,47^ which have made the collection of high-resolution data sets possible worldwide. Google and Aclima already have data for other cities such as London, Amsterdam, and Copenhagen as part of the Air View Project.^60^ Other initiatives such as those from the MIT Senseable City Laboratory^37,39,88^ have been developing and deploying sensing platforms on vehicles, such as waste collection vehicles, to collect large-scale environmental data in cities. Additionally, GSV imagery is available globally, with over 220 billion GSV images in more than 100 countries, which may be used to produce urban greenspace and other metrics.^62^

As the precise mechanisms of pollution reduction through urban greenspace are still unclear,^17^ this warrants further investigation in the future. Further research on dry deposition, dispersion, and ecophysiological processes linked to greenspace should be conducted. Future research could also include urban morphology among the control variables since the size, structure, and growth of cities can affect air quality in urban settings.^7^ In addition, forthcoming work could use the DTP method^89^ to calculate the GVI. This is a segmentation method that applies vision transformers to images and produces more detailed greenspace metric outputs. DTP is an excellent way of computing urban street-level greenspace metrics; however, in this study, we used an image segmentation computer vision methodology, which has been broadly used until recently.^45,50,90,91^ Our GVI metrics were modeled before the DTP methodology^89^ was published. Moreover, future research should also analyze disparities in air pollution exposures and access to and exposures to urban greenspace in Dublin City. In relation to these issues, Venter et al.^92^ in Oslo, Norway, reported that environmental equity has been neglected in one of the most affluent cities in the world. Wang et al.^93^ showed that ethnic minorities in the United States are unfairly exposed to higher levels of air pollutants in comparison to the white population, and Rehling et al.^94^ presented evidence that children of lower socio-economic status need to travel larger distances to access greenspaces in Germany, relative to more affluent people. These results evoke an interest in understanding how positive and negative exposure inequities develop and exist in urban areas in nations globally, and our results can help to inform this research. Finally, this research can help leaders, policymakers, and governments shape their efforts toward smart, greener, healthy, equitable, and sustainable future cities.

## 5 Implications

This study examined associations between urban greenspace and pollution where both have been resolved in extremely high resolution and accuracy over the entire road network of a major city. Air pollution was quantified and mapped using 5,030,143 Google Air View point measurements, and urban greenspace was quantified using 403,409 Google Street View images combined with computer vision methods. Urban greenspace was also determined using satellite imagery and computational algorithms. After controlling for meteorology, traffic, and population density, the study indicated statistically significant (*p* < 0.001) associations between higher levels of urban greenspace (GVI, GVI:NDVI, and NDVI) and decreased pollution levels (PM_2.5_, NO_2_, NO, CO_2_, and CO) in almost all scenarios studied, with a few minor exceptions. The study provides further insights into how large-scale digital data can be harnessed to monitor urban environmental metrics and interactions in detail and at scale in cities globally.^95−98^ The results acknowledge the inverse association between urban greenspace and air pollution in urban areas and, consequently, their potential to help improve public health. As we urgently need to accellerate progress towards the United Nations Agenda 2030 Sustainable Development Goals and align research and innovation with this aim,^99^ this study closely aligns with Goal 11 related to sustainable cities and communities and Goal 3 related to good health and wellbeing. In a world which is rapidly urbanizing, governments and municipalities must adopt and refine strategies for improving environmental health in urban spaces in order to create more sustainable, greener, and healthy cities for all.

## Supporting Information Available

Auxiliary tables for Moran’s *I*, Lagrange multiplier, and robust LM tests; figures on high-resolution version of greenspace and air pollutants; spatial lag regression coefficients table; correlation tables; graphs of Google Air View vs EPA measurements; and scatterplots for actual values vs predicted values for all models (PDF)
