# Supplementary material for: Data Insights for Sustainable Cities: Associations between Google Street View-Derived Urban Greenspace and Google Air View-Derived Pollution Levels
Source: Environ Sci Technol. 2023 Nov 16;57(48):19637–48. doi: 10.1021/acs.est.3c05000 (PMC10702516; doi:10.1021/acs.est.3c05000)
Supplement: Supplementary file 1 — es3c05000_si_001.pdf [file es3c05000_si_001.pdf]

Supporting Information for:

Data Insights for Sustainable Cities: Associations between Google Street View-derived Urban Greenspace & Google Air View-derived Pollution Levels

Maria E. S. Sabedotti[a,c,1], Anna C. O'Regan[a,b,c], Marguerite M. Nyhan[a,b,c,1,*]

[a]*Discipline of Civil, Structural & Environmental Engineering, School of Engineering & Architecture, University College Cork, Cork, Ireland.*

[b]*MaREI, the SFI Research Centre for Energy, Climate & Marine, University College Cork, Ringaskiddy, Cork, P43 C573, Ireland.*

[c]*Environmental Research Institute, University College Cork, Lee Rd, Sunday's Well, Cork, T23 XE10, Ireland*

[1]Co-first authors. These authors contributed equally to this work.

*Corresponding Author: Dr. Marguerite M. Nyhan. Email: marguerite.nyhan@ucc.ie

Pages: 38

Figures: 29

Tables: 8

Table S1. Moran's I test for pollutants ($PM_{2.5}$, NO_2, NO, CO, and CO_2) for all spatial scenarios studied (all point locations and for buffer zones of radius 100m, 300m, 500m, 1000m, and 2000m) (n=24,694). Pollutants with results over 0.3 were considered spatially auto-correlated.

Pollutant	Points	100m	300m	500m	1000m	2000m
**$PM_{2.5}$**	0.570**	0.547**	0.551**	0.551**	0.554**	0.563**
**NO_2**	0.543**	0.541**	0.548**	0.548**	0.550**	0.551**
**NO**	0.339**	0.343**	0.350**	0.350**	0.357**	0.359**
**CO**	0.698**	0.692**	0.692**	0.692**	0.693**	0.697**
**CO_2**	0.654**	0.653**	0.652**	0.652**	0.649**	0.651**

* *p<0.05*
** *p <0.001*

Table S2. Lagrange Multiplier and Robust LM tests for all models analysing associations between urban greenspace (GVI, NDVI, GVI:NDVI) and $PM_{2.5}$, for all spatial scenarios studied (all point locations and for buffer zones of radius 100m, 300m, 500m, 1000m, and 2000m) (n=24,694).

Pollutant	Test	Points	100m	300m	500m	1000m	2000m
**GVI**	**LM_{lag}**	1.74E+04**	1.72E+04**	1.79E+04**	1.79E+04**	1.81E+04**	1.85E+04**
	**LM_{err}**	1.77E+04**	1.30E+02**	1.82E+04**	1.82E+04**	1.84E+04**	1.88E+04**
	**R LM_{lag}**	1.43E+02**	1.74E+04**	1.06E+02**	1.05E+02**	1.15E+02**	1.17E+02**
	**R LM_{err}**	3.96E+02**	3.74E+02**	4.02E+02**	4.02E+02**	4.19E+02**	4.33E+02**
**NDVI**	**LM_{lag}**	1.66E+04**	1.60E+04**	1.66E+04**	1.66E+04**	1.70E+04**	1.77E+04**
	**LM_{err}**	2.99E+02**	1.62E+04**	1.68E+04**	1.66E+04**	1.72E+04**	1.80E+04**
	**R LM_{lag}**	1.66E+04**	1.46E+02**	1.21E+02**	1.20E+02**	1.51E+02**	1.52E+02**
	**R LM_{err}**	3.25E+02**	3.22E+02**	3.47E+02**	3.46E+02**	3.66E+02**	3.88E+02**
**GVI:NDVI**	**LM_{lag}**	1.75E+04**	1.72E+04**	3.47E+02**	1.79E+04**	1.82E+04**	1.86E+04**
	**LM_{err}**	1.77E+04**	1.26E+02**	1.83E+04**	1.82E+04**	1.85E+04**	1.89E+04**
	**R LM_{lag}**	1.36E+02**	1.75E+04**	1.03E+02**	1.02E+02**	1.13E+02**	1.15E+02**
	**R LM_{err}**	1.79E+04**	3.75E+02**	4.04E+02**	4.03E+02**	4.21E+02**	4.34E+02**

* $p<0.05$
** $p<0.001$

Table S3. Lagrange Multiplier and Robust LM tests for all models analysing associations between urban greenspace (GVI, NDVI, GVI:NDVI) and NO_2, for all spatial scenarios studied (all point locations and for buffer zones of radius 100m, 300m, 500m, 1000m, and 2000m) (n=24,694).

Pollutant	Test	Points	100m	300m	500m	1000m	2000m
**GVI**	**LM_{lag}**	1.11E+04**	1.17E+04**	1.20E+04**	1.20E+04**	1.14E+04**	1.14E+04**
	**LM_{err}**	1.12E+04**	1.17E+04**	1.20E+04**	1.20E+04**	1.12E+04**	1.13E+04**
	**R LM_{lag}**	3.44E+02**	4.13E+02**	4.19E+02**	4.17E+02**	4.88E+02**	4.72E+02**
	**R LM_{err}**	3.76E+02**	3.29E+02**	3.91E+02**	3.92E+02**	3.50E+02**	3.66E+02**
**NDVI**	**LM_{lag}**	1.02E+04**	1.04E+04**	1.08E+04**	1.08E+04**	1.09E+04**	1.11E+04**
	**LM_{err}**	9.92E+03**	1.03E+04**	1.07E+04**	1.08E+04**	1.07E+04**	1.10E+04**
	**R LM_{lag}**	5.52E+02**	4.16E+02**	4.25E+02**	4.24E+02**	5.13E+02**	4.84E+02**
	**R LM_{err}**	2.69E+02**	2.99E+02**	3.51E+02**	3.52E+02**	3.17E+02**	3.46E+02**
**GVI:NDVI**	**LM_{lag}**	1.12E+04**	1.18E+04**	1.21E+04**	1.21E+04**	1.14E+04**	1.14E+04**
	**LM_{err}**	1.12E+04**	1.17E+04**	1.20E+04**	1.21E+04**	1.13E+04**	1.13E+04**
	**R LM_{lag}**	3.33E+02**	4.04E+02**	4.13E+02**	4.11E+02**	4.84E+02**	4.68E+02**
	**R LM_{err}**	3.83E+02**	3.33E+02**	3.94E+02**	3.95E+02**	3.53E+02**	3.69E+02**

* $p<0.05$
** $p<0.001$

Table S4. Lagrange Multiplier and Robust LM tests for all models analysing associations between urban greenspace (GVI, NDVI, GVI:NDVI) and NO, for all spatial scenarios studied (all point locations and for buffer zones of radius 100m, 300m, 500m, 1000m, and 2000m) (n=24,694).

Pollutant	Test	Points	100m	300m	500m	1000m	2000m
GVI	LM_{lag}	2.76E+03**	2.99E+03**	3.24E+03**	3.24E+03**	3.27E+03**	3.33E+03**
	LM_{err}	2.67E+03**	2.86E+03**	3.11E+03**	3.11E+03**	3.13E+03**	3.20E+03**
	R LM_{lag}	9.38E+01**	1.26E+02**	1.27E+02**	1.27E+02**	1.42E+02**	1.34E+02**
	R LM_{err}	3.54E+00	1.63E-01	2.00E-01	2.19E-01	1.95E-01	4.52E-01
NDVI	LM_{lag}	2.62E+03**	2.74E+03**	3.06E+03**	3.06E+03**	3.23E+03**	3.31E+03**
	LM_{err}	2.49E+03**	2.66E+03**	2.95E+03**	1.09E+02**	3.09E+03**	3.18E+03**
	R LM_{lag}	1.28E+02**	9.01E+01**	1.08E+02**	2.95E+03**	1.35E+02**	1.32E+02**
	R LM_{err}	9.60E-01	3.69E+00	6.48E-01	6.68E-01	2.63E-01	4.47E-01
GVI:NDVI	LM_{lag}	2.78E+03**	3.00E+03**	3.25E+03**	3.25E+03**	3.28E+03**	3.33E+03**
	LM_{err}	2.69E+03**	1.27E+02**	3.13E+03**	1.27E+02**	3.13E+03**	3.20E+03**
	R LM_{lag}	9.26E+01**	2.87E+03**	1.27E+02**	3.12E+03**	1.42E+02**	1.34E+02**
	R LM_{err}	3.50E+00	1.11E-01	2.01E-01	2.19E-01	1.91E-01	4.70E-01

* *p<0.05*

** *p <0.001*

Table S5. Lagrange Multiplier and Robust LM tests for all models analysing associations between urban greenspace (GVI, NDVI, GVI:NDVI) and CO, for all spatial scenarios studied (all point locations and for buffer zones of radius 100m, 300m, 500m, 1000m, and 2000m) (n=24,694).

Pollutant	Test	Points	100m	300m	500m	1000m	2000m
**GVI**	**LM_{lag}**	2.13E+04**	2.27E+04**	2.35E+04**	2.36E+04**	2.36E+04**	2.37E+04**
	**LM_{err}**	2.20E+04**	2.33E+04**	2.43E+04**	2.43E+04**	2.43E+04**	2.45E+04**
	**R LM_{lag}**	5.53E+02**	6.06E+02**	5.06E+02**	5.06E+02**	4.82E+02**	4.57E+02**
	**R LM_{err}**	1.17E+03**	1.20E+03**	1.24E+03**	1.23E+03**	1.25E+03**	1.31E+03**
**NDVI**	**LM_{lag}**	1.97E+04**	2.10E+04**	2.26E+04**	2.26E+04**	2.32E+04**	2.32E+04**
	**LM_{err}**	2.00E+04**	2.16E+04**	2.33E+04**	2.33E+04**	2.40E+04**	2.40E+04**
	**R LM_{lag}**	8.17E+02**	5.54E+02**	4.77E+02**	4.77E+02**	4.71E+02**	4.42E+02**
	**R LM_{err}**	1.05E+03**	1.13E+03**	1.20E+03**	1.19E+03**	1.24E+03**	1.29E+03**
**GVI:NDVI**	**LM_{lag}**	2.14E+04**	2.28E+04**	2.36E+04**	2.36E+04**	2.36E+04**	2.37E+04**
	**LM_{err}**	2.20E+04**	2.34E+04**	2.43E+04**	2.43E+04**	2.44E+04**	2.46E+04**
	**R LM_{lag}**	5.40E+02**	5.97E+02**	2.43E+04**	5.02E+02**	4.81E+02**	4.57E+02**
	**R LM_{err}**	1.17E+03**	1.20E+03**	1.23E+03**	1.23E+03**	1.25E+03**	1.31E+03**

* *p<0.05*
** *p <0.001*

Table S6. Lagrange Multiplier and Robust LM tests for all models analysing associations between urban greenspace (GVI, NDVI, GVI:NDVI) and CO_2, for all spatial scenarios studied (all point locations and for buffer zones of radius 100m, 300m, 500m, 1000m, and 2000m) (n=24,694).

Pollutant	Test	Points	100m	300m	500m	1000m	2000m
GVI	LM_{lag}	2.31E+04**	2.43E+04**	2.51E+04**	2.51E+04**	2.50E+04**	2.50E+04**
	LM_{err}	2.34E+04**	2.44E+04**	2.54E+04**	2.53E+04**	2.53E+04**	2.54E+04**
	R LM_{lag}	9.50E+02**	1.04E+03**	9.20E+02**	9.22E+02**	9.40E+02**	9.32E+02**
	R LM_{err}	1.23E+03**	1.12E+03**	1.14E+03**	1.14E+03**	1.27E+03**	1.31E+03**
NDVI	LM_{lag}	2.18E+04**	2.28E+04**	2.38E+04**	2.38E+04**	2.41E+04**	2.41E+04**
	LM_{err}	2.17E+04**	2.27E+04**	2.39E+04**	2.39E+04**	2.43E+04**	2.43E+04**
	R LM_{lag}	1.20E+03**	1.06E+03**	9.81E+02**	9.84E+02**	1.00E+03**	9.90E+02**
	R LM_{err}	1.12E+03**	1.01E+03**	1.06E+03**	1.06E+03**	1.20E+03**	1.23E+03**
GVI:NDVI	LM_{lag}	2.31E+04**	2.44E+04**	2.52E+04**	2.51E+04**	2.50E+04**	2.50E+04**
	LM_{err}	2.34E+04**	2.45E+04**	2.54E+04**	2.54E+04**	2.53E+04**	2.54E+04**
	R LM_{lag}	9.36E+02**	1.03E+03**	9.09E+02**	9.11E+02**	9.35E+02**	9.27E+02**
	R LM_{err}	1.24E+03**	1.12E+03**	1.14E+03**	1.14E+03**	1.28E+03**	1.32E+03**

* $p<0.05$
** $p<0.001$

Table S7. Moran's I test for the residuals of the Spatial Lag regression analysis for all models analysing associations between urban greenspace (GVI, NDVI, GVI:NDVI) and air pollution ($PM_{2.5}$, NO_2, NO, CO, and CO_2) levels. Models analysed these associations for all for all spatial scenarios studied (all point locations and for buffer zones of radius 100m, 300m, 500m, 1000m, and 2000m) (n=24,694). All models were adjusted for traffic, precipitation, temperature and population density.

**Pollutant**	**Urban greenspace metric**	**Points**	**100m**	**300m**	**500m**	**1000m**	**2000m**
**ln($PM_{2.5}$)**	GVI	0.058**	0.081**	0.104**	0.104**	0.097**	0.098**
	NDVI	-0.020**	0.039**	0.056**	0.055**	0.047**	0.047**
	GVI:NDVI	0.064**	0.085**	0.108**	0.108**	0.100**	0.101**
**ln(NO_2)**	GVI	-0.017**	-0.040**	-0.022**	-0.021**	-0.034**	-0.030**
	NDVI	-0.049**	-0.033**	-0.025**	-0.025**	-0.040**	-0.036**
	GVI:NDVI	-0.013*	-0.039**	-0.021**	-0.020**	-0.033**	-0.029**
**ln(NO)**	GVI	-0.075**	-0.115**	-0.116**	-0.116**	-0.115**	-0.114**
	NDVI	-0.082**	-0.100**	-0.116**	-0.116**	-0.114**	-0.115**
	GVI:NDVI	-0.077**	-0.115**	-0.116**	-0.116**	-0.116**	-0.114**
**ln(CO)**	GVI	0.076**	0.070**	0.092**	0.091**	0.093**	0.107**
	NDVI	0.027**	0.075**	0.094**	0.094**	0.096**	0.156**
	GVI:NDVI	0.079**	0.072**	0.092**	0.092**	0.093**	0.107**
**ln(CO_2)**	GVI	0.019**	-0.006	-0.004	-0.004	0.011*	0.016**
	NDVI	-0.008*	-0.014**	-0.015**	-0.015**	-0.001	0.003
	GVI:NDVI	0.021**	-0.005	-0.003	-0.003	0.011*	0.017**

* *p<0.05*
** *p <0.001*

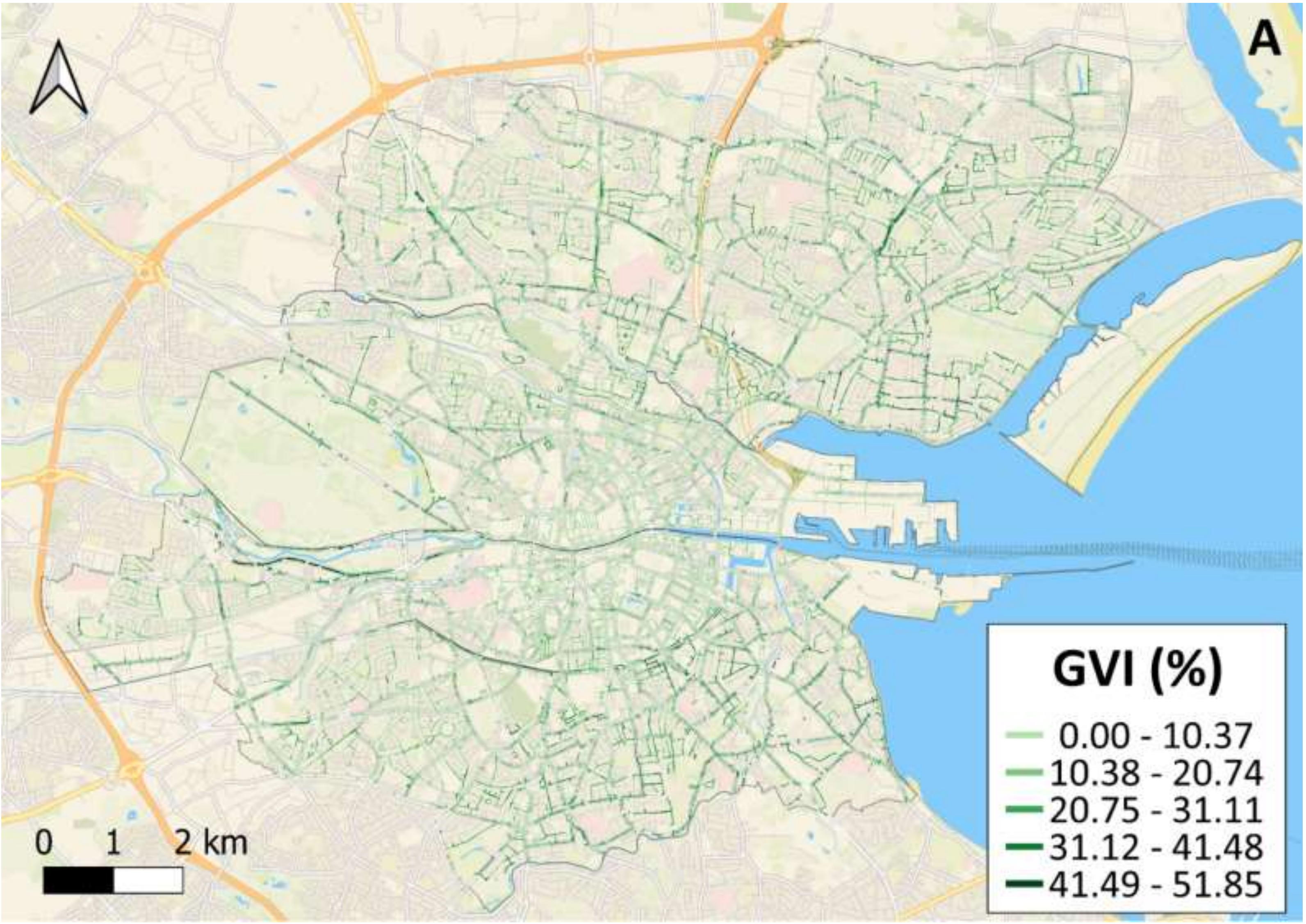

Figure S1. High-resolution version of figure 1a in the main manuscript: Map of urban greenspace Green View Index (GVI) computed in high spatial resolution (67,265 point locations) on the entire road network of Dublin City.

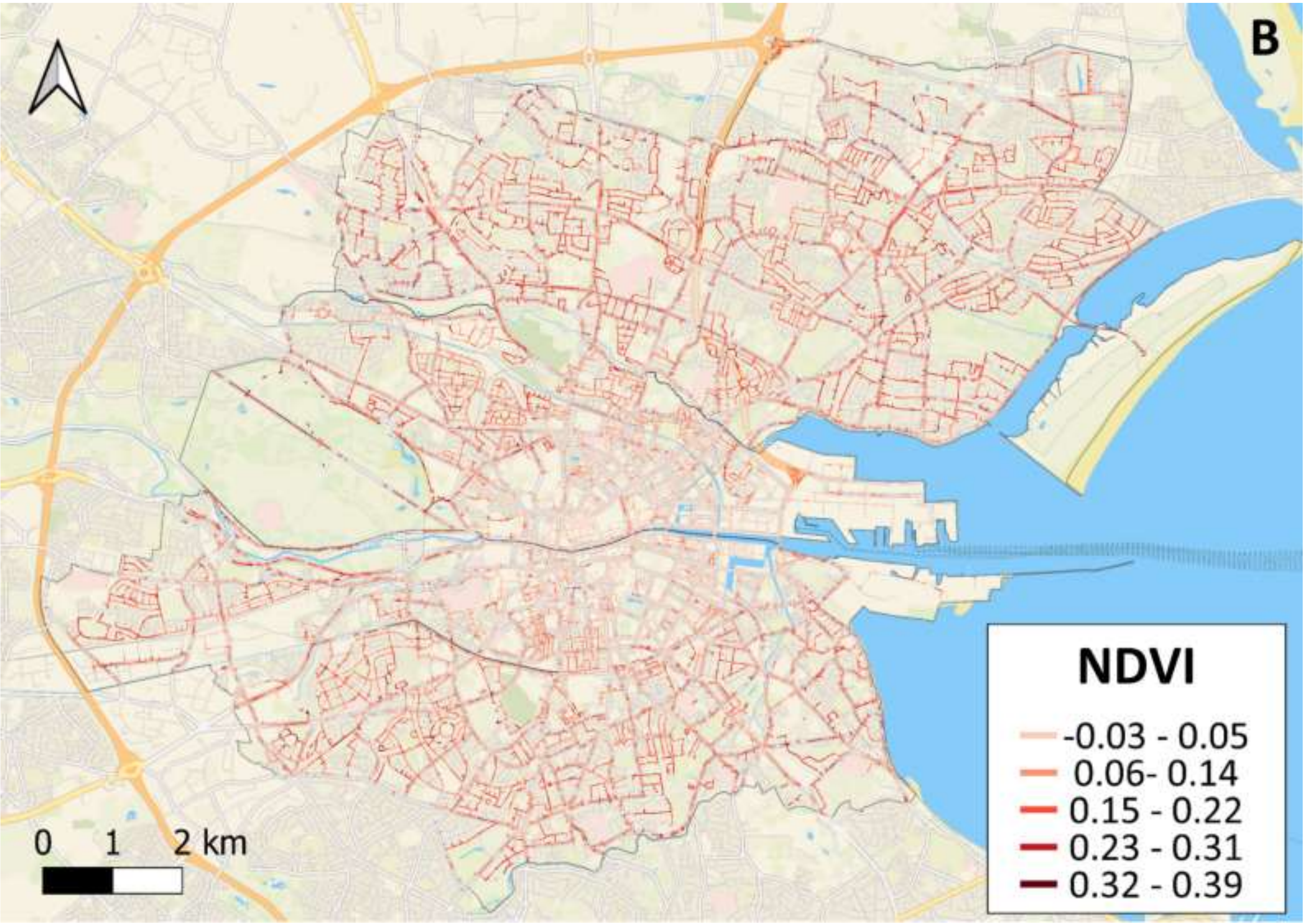

Figure S2. High-resolution version of figure 1b in the main manuscript. Map of urban greenspace Normalised Difference Vegetation Index (NDVI) computed in high spatial resolution (67,265 point locations) on the entire road network of Dublin City.

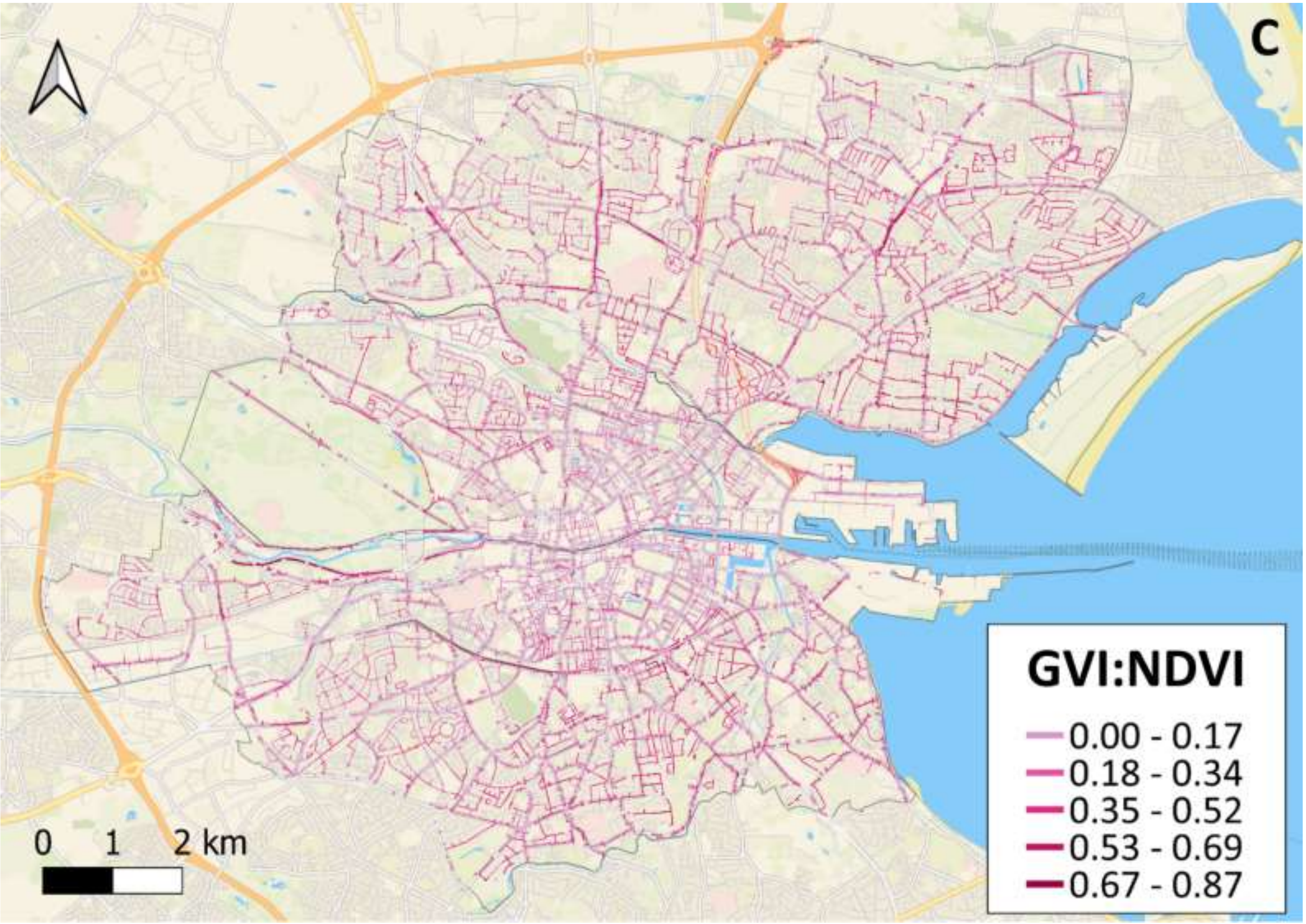

Figure S3. High-resolution version of figure 1c in the main manuscript. Map of urban greenspace metric (GVI:NDVI) computed in high spatial resolution (67,265 point locations) on the entire road network of Dublin City.

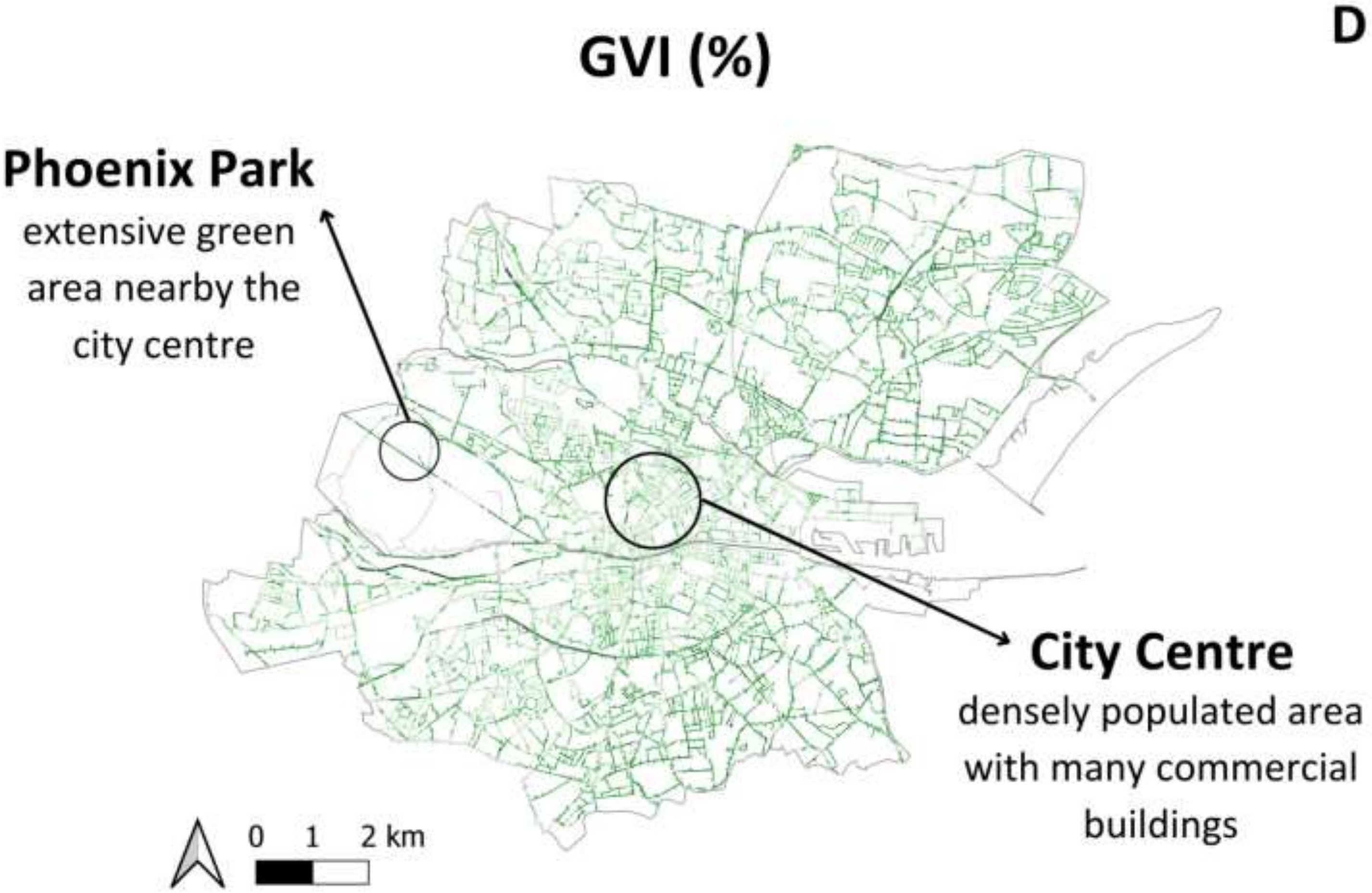

Figure S4. High-resolution version of figure 1d in the main manuscript. The urban greenspace Green View Index (GVI) (map D) highlights specific sites representing a dense urban City Centre area and a major urban park (Phoenix Park) which assist in the visual interpretation of the urban greenspace metrics computed across the city.

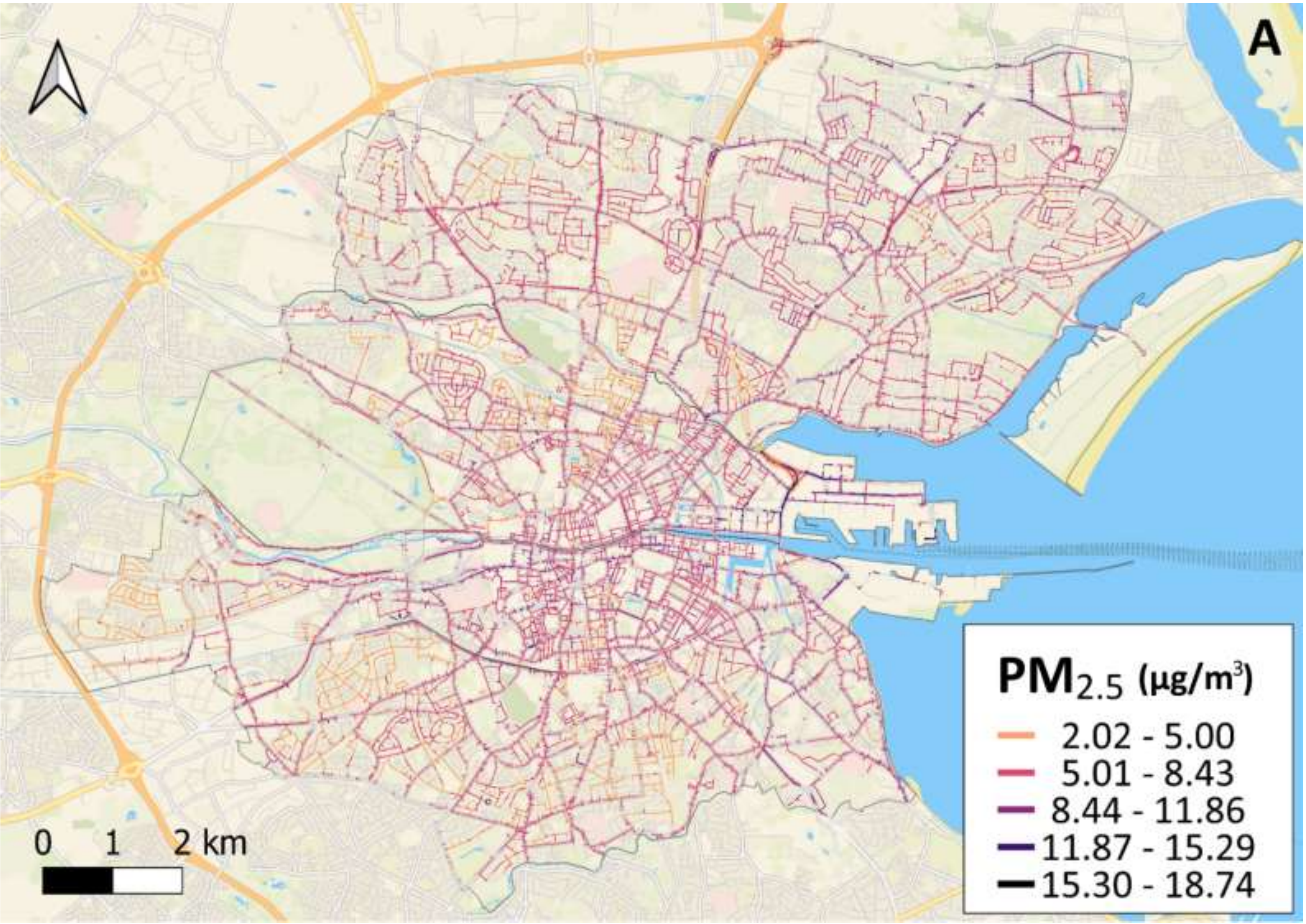

Figure S5. High-resolution version of figure 2a in the main manuscript. Map of Google Air View-derived $PM_{2.5}$ air pollution quantified in high spatial resolution using 5,030,143 point measurements on 24,694 road links (50m road segments) across the entire road network of Dublin City.

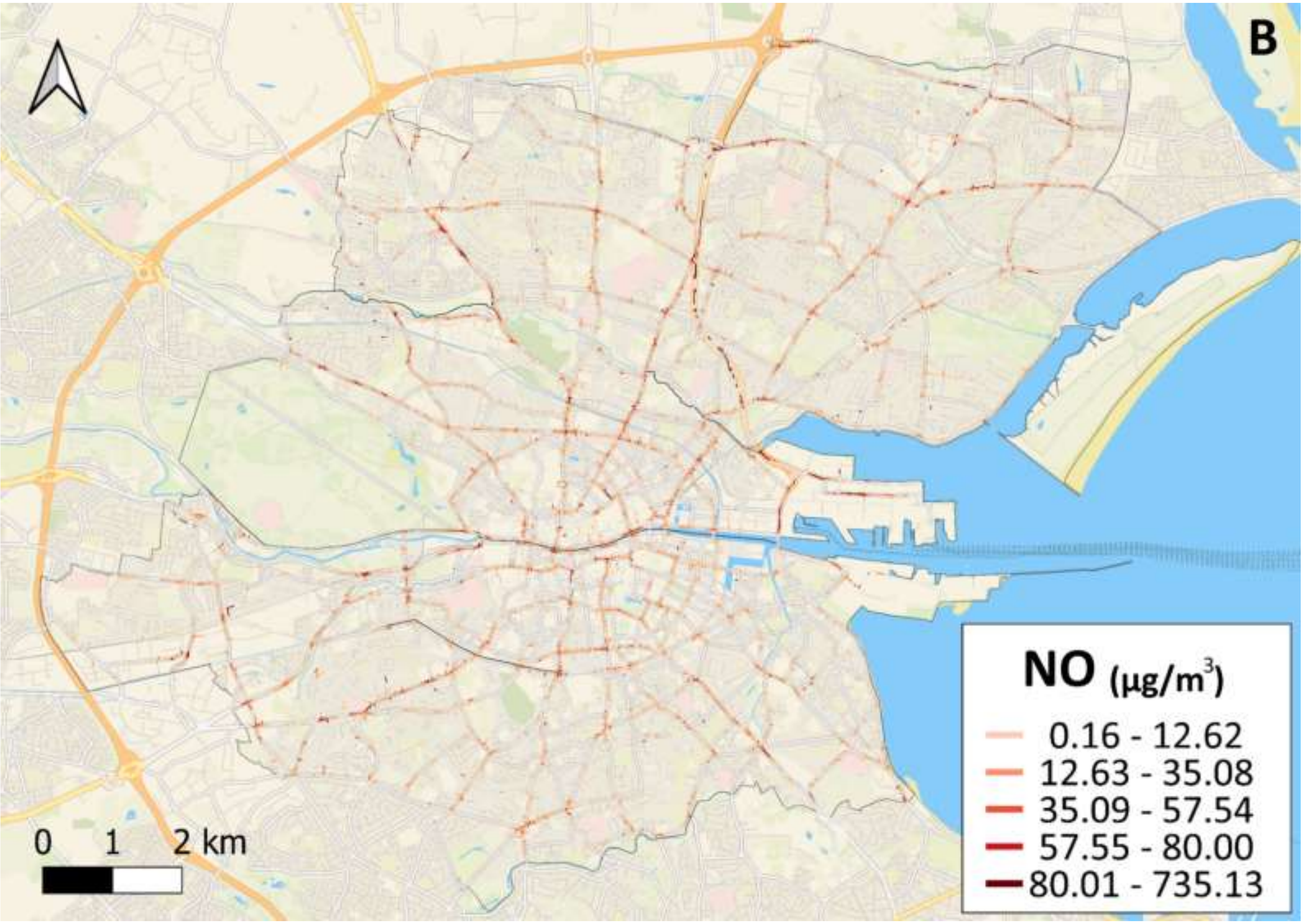

Figure S6. High-resolution version of Figure 2b in the main manuscript. Map of Google Air View-derived NO air pollution quantified in high spatial resolution using 5,030,143 point measurements on 24,694 road links (50m road segments) across the entire road network of Dublin City.

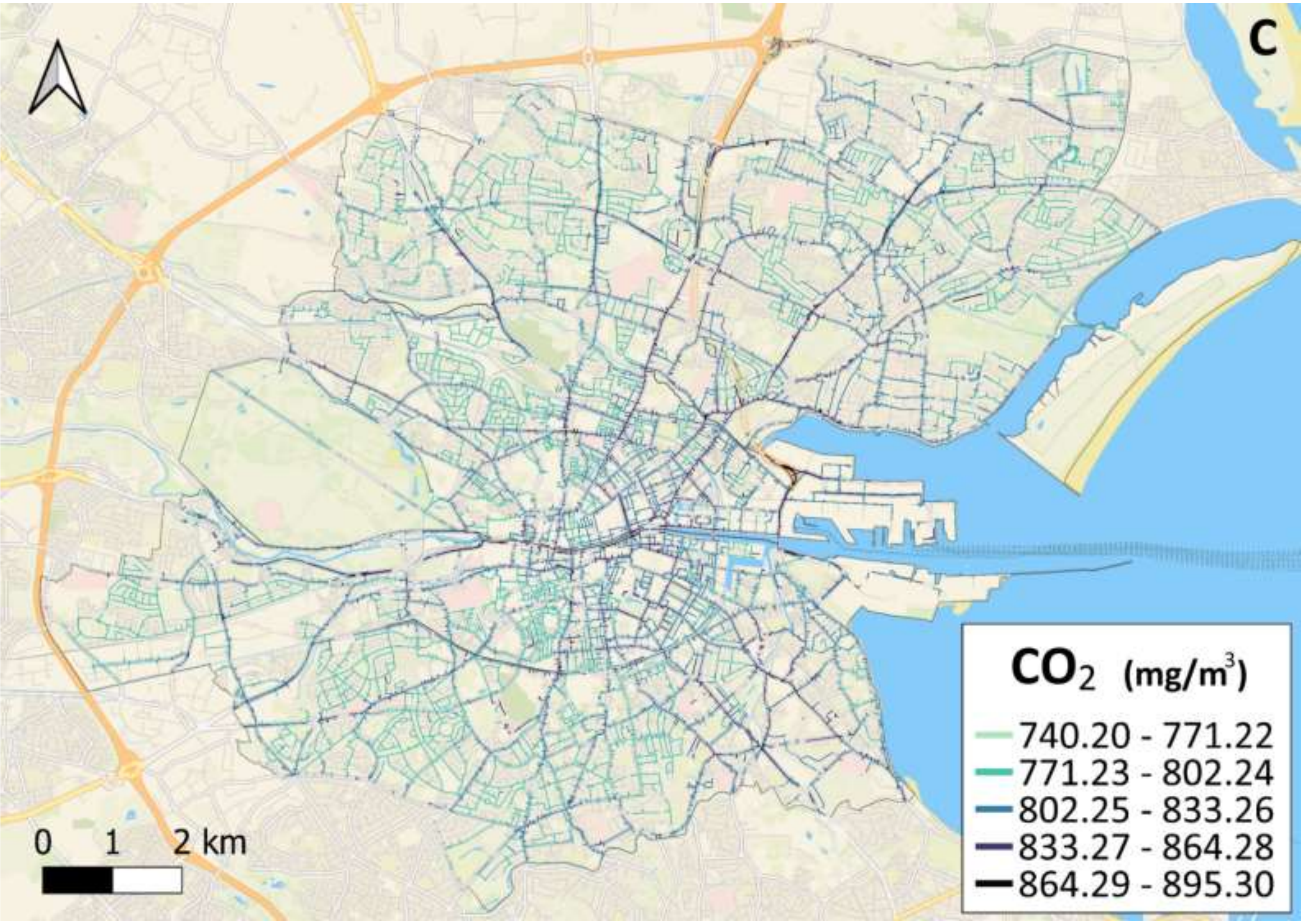

Figure S7. High-resolution version of figure 2c in the main manuscript. Map of Google Air View CO_2 quantified in high spatial resolution using 5,030,143 point measurements on 24,694 road links (50m road segments) across the entire road network of Dublin City.

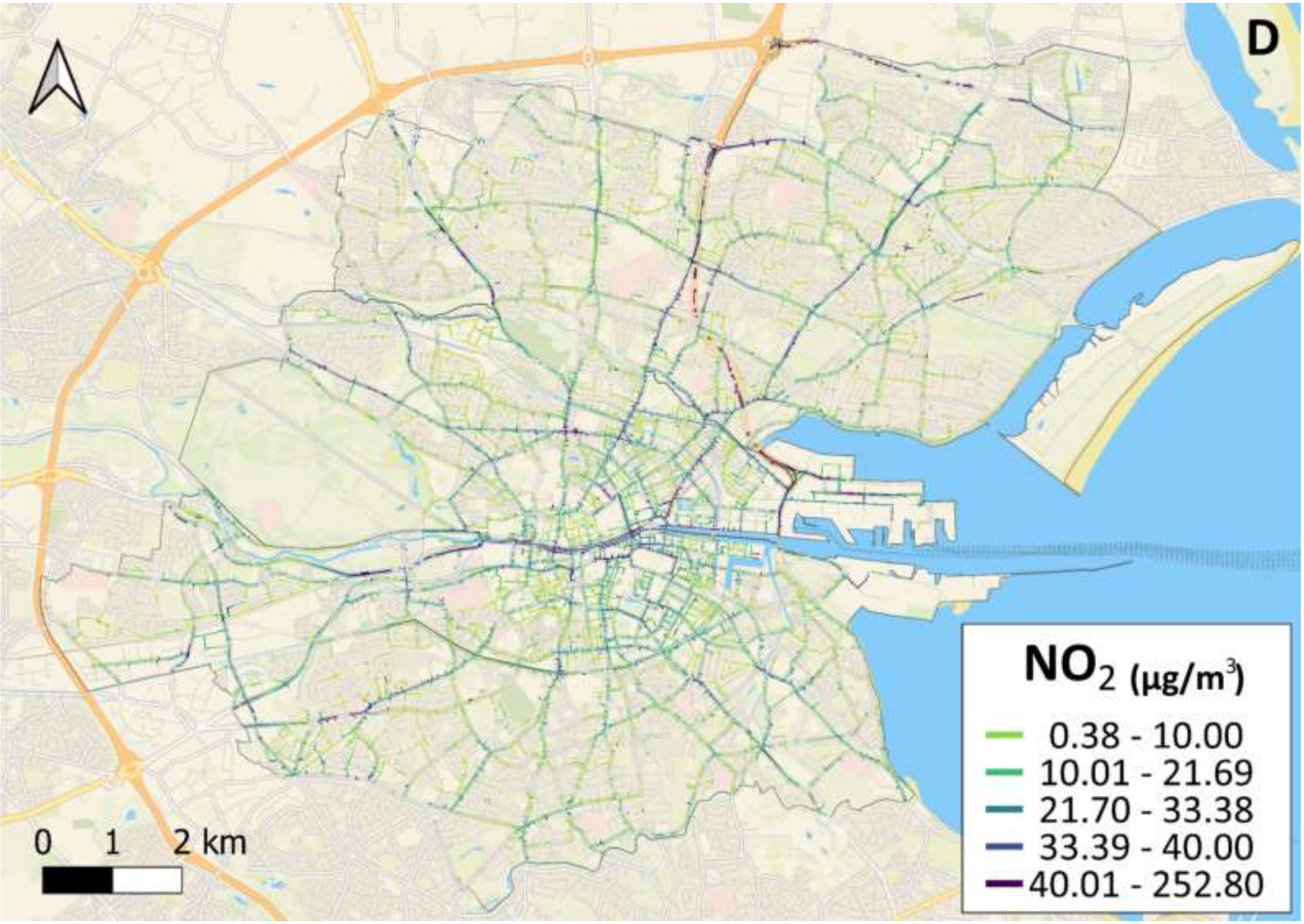

Figure S8. High-resolution version of Figure 2d in the main manuscript. Map of Google Air View-derived NO_2 air pollution quantified in high spatial resolution using 5,030,143 point measurements on 24,694 road links (50m road segments) across the entire road network of Dublin City.

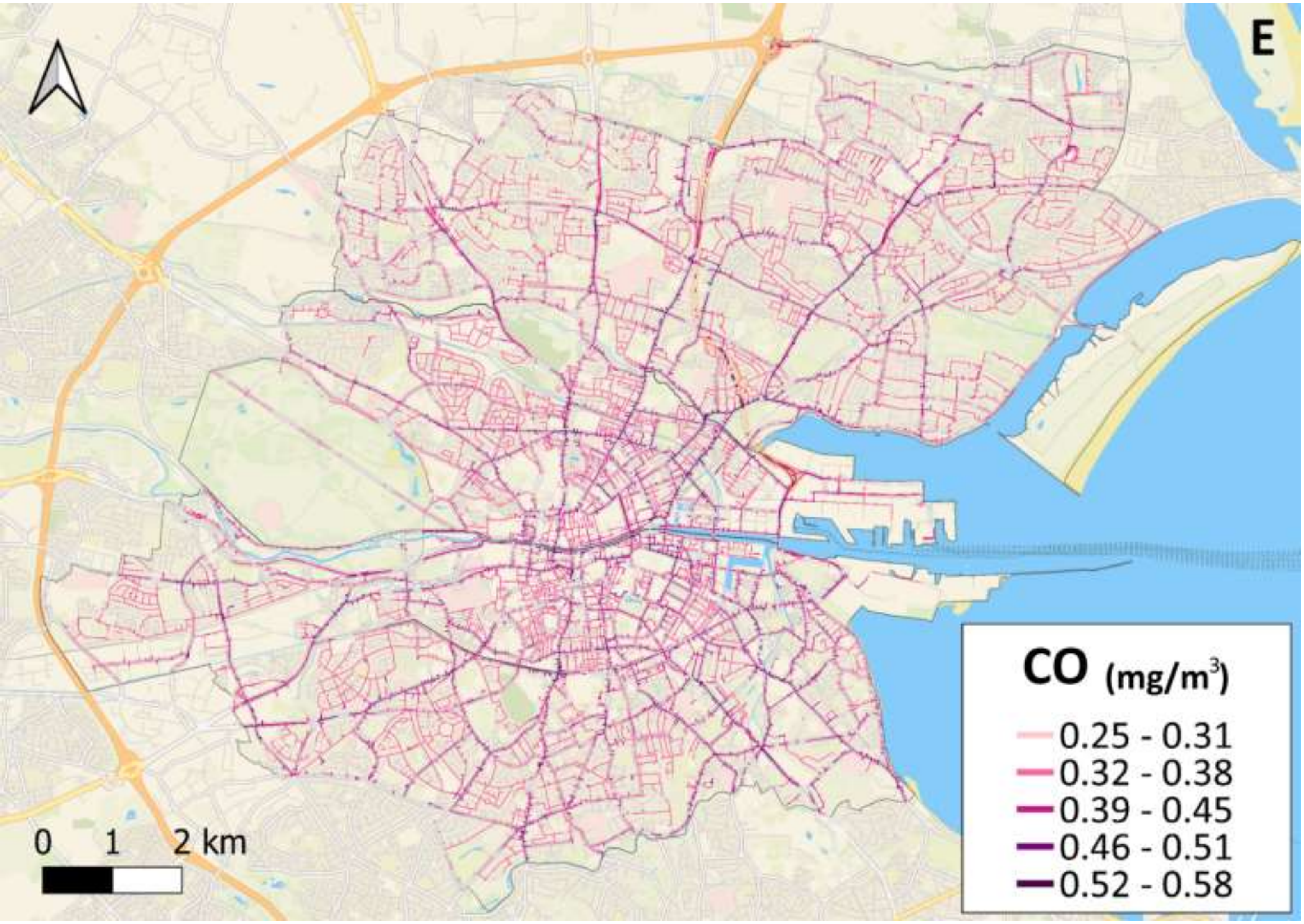

Figure S9. High-resolution version of Figure 2e in the main manuscript. Map of Google Air View-derived CO air pollution quantified in high spatial resolution using 5,030,143 point measurements on 24,694 road links (50m road segments) across the entire road network of Dublin City.

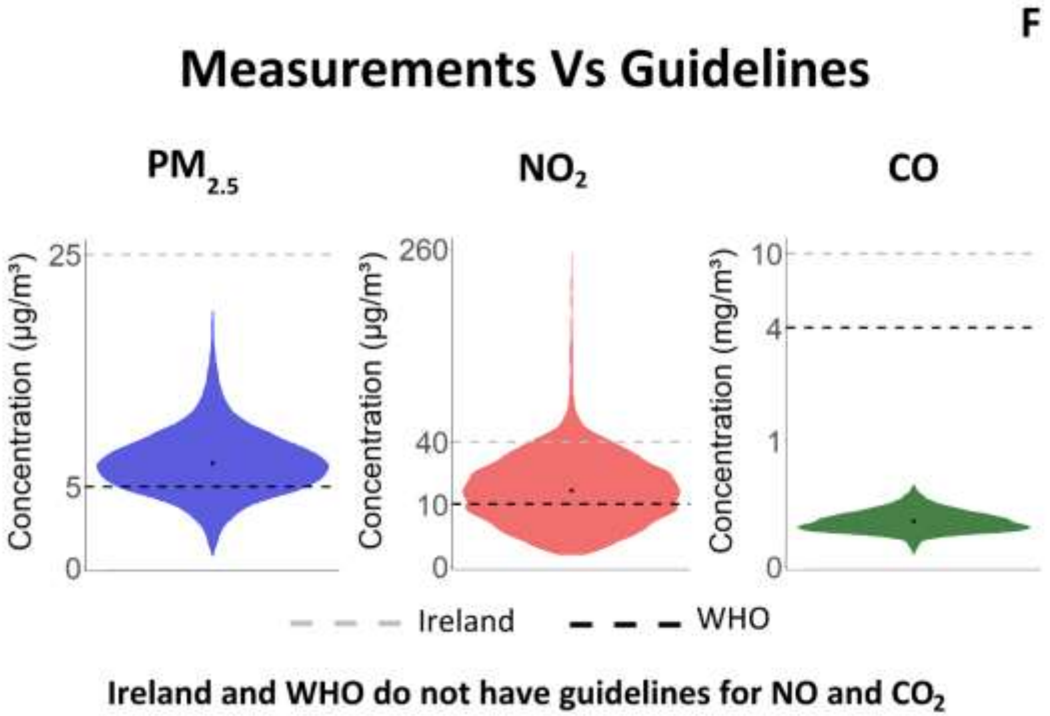

Figure S10. High-resolution version of Figure 2f in the main manuscript. Graphs show comparisons of citywide $PM_{2.5}$, NO_2 and CO pollution distributions with national Irish and international World Health Organization limits.

Table S8. Spatial lag Regression coefficients for all models analysing associations between urban greenspace (GVI, NDVI, GVI:NDVI) and air pollution ($PM_{2.5}$, NO_2, NO, CO and CO_2) levels. Models analysed these associations for point locations and all buffer zones of radius 100m, 300m, 500m, 1000m, and 2000m (n=24,694). All models were adjusted for traffic, precipitation, temperature and population density. Note that these results consider the natural log of all pollution dependent variables.

Pollutant	Urban greenspace metric	Points	100m	300m	500m	1000m	2000m
		x 10^{-2}					
$\ln(PM_{2.5})$	GVI	-0.065	-0.232**	-0.379**	-0.376**	-0.598**	-0.592**
	NDVI	-72.473**	-107.555**	-144.406**	-144.132**	-183.766**	-206.959**
	GVI:NDVI	-2.245	-11.880**	-20.704**	-20.571**	-34.214**	-32.398**
$\ln(NO_2)$	GVI	-0.532*	-1.334**	-2.661**	-2.629**	-4.987**	-7.161**
	NDVI	-366.510**	-531.854**	-670.451**	-667.546**	-837.716**	-1073.719**
	GVI:NDVI	-25.124	-70.618**	-151.790**	-149.781**	-299.963**	-431.706**
ln(NO)	GVI	-2.259**	-2.845*	-2.741	-2.775	-4.051	-4.018
	NDVI	-446.110**	-583.520*	-547.109	-547.868	-536.110	-596.546
	GVI:NDVI	-127.628**	-162.197*	-159.179	-161.302	-249.159	-241.964
ln(CO)	GVI	-0.098**	-0.120**	-0.122**	-0.122**	-0.177**	-0.252**
	NDVI	-46.436**	-56.237**	-49.327**	-49.303**	-46.347**	-72.281**
	GVI:NDVI	-4.509**	-5.723**	-6.131**	-6.117**	-9.763**	-13.405**
$\ln(CO_2)$	GVI	-0.011*	-0.015*	-0.013	-0.013	-0.031*	-0.055**
	NDVI	-7.725**	-9.862**	-11.184**	-11.148**	-12.637**	-17.929**
	GVI:NDVI	-0.455	-0.645	-0.515	-0.496	-1.689*	-3.048**

* $p<0.05$
** $p<0.001$

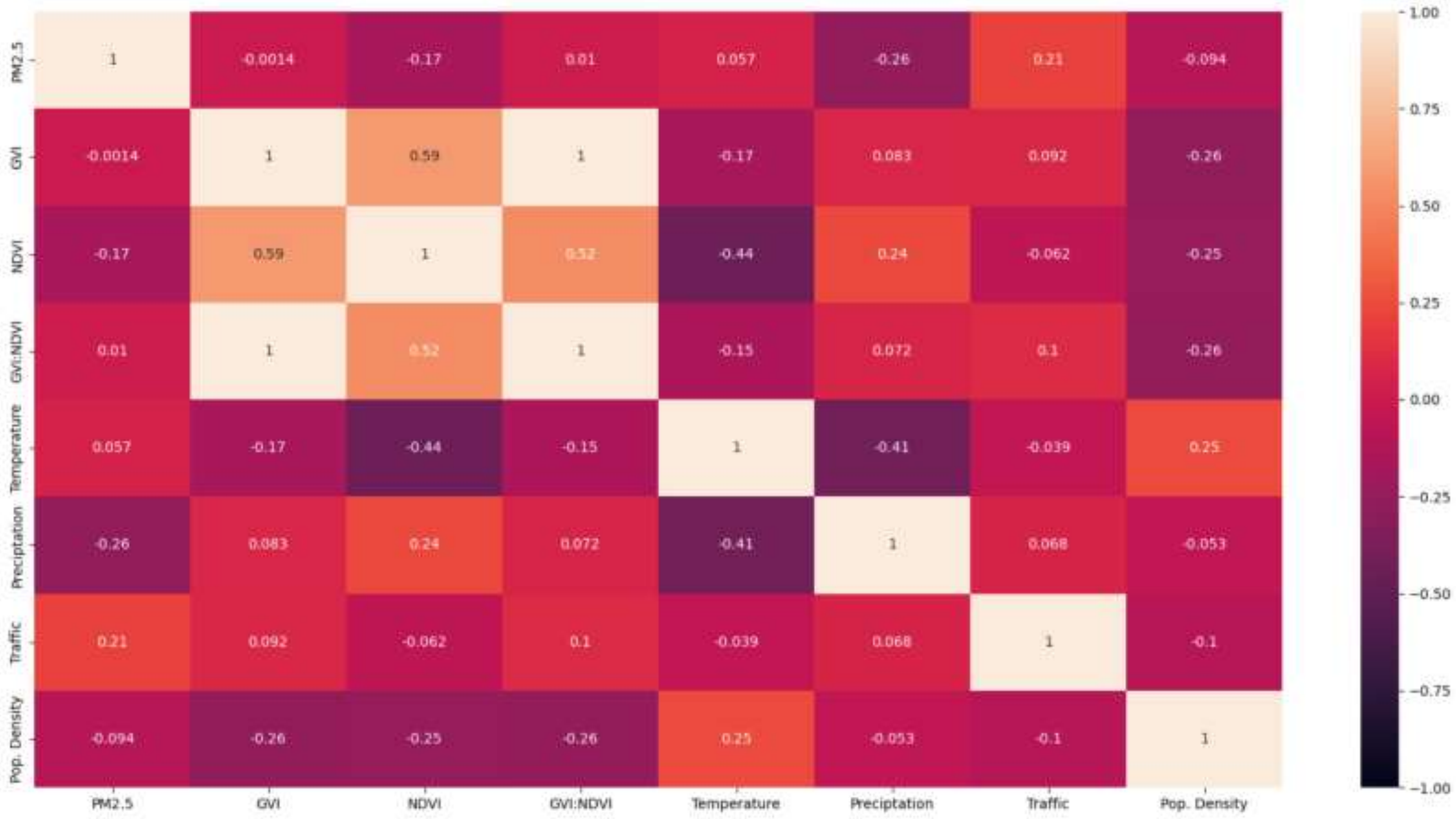

Figure S11. Correlation table for $PM_{2.5}$, all the urban greenspace metrics (GVI, NDVI, GVI:NDVI), and control variables (temperature, precipitation, traffic and population density), for all point locations (n=24,694).

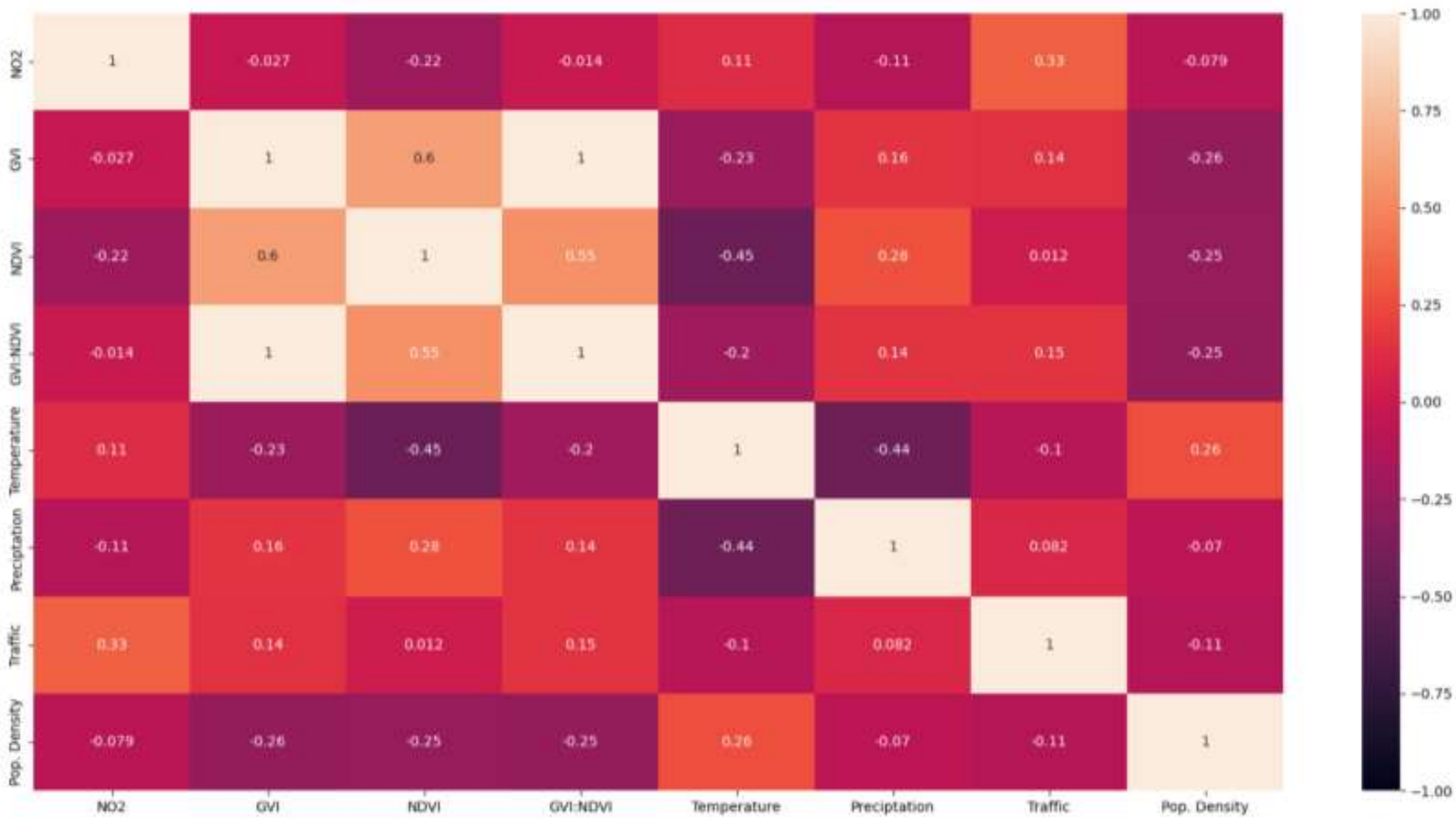

Figure S12. Correlation table for NO_2, all the urban greenspace metrics (GVI, NDVI, GVI:NDVI), and control variables (temperature, precipitation, traffic and population density), for all point locations (n=24,694).

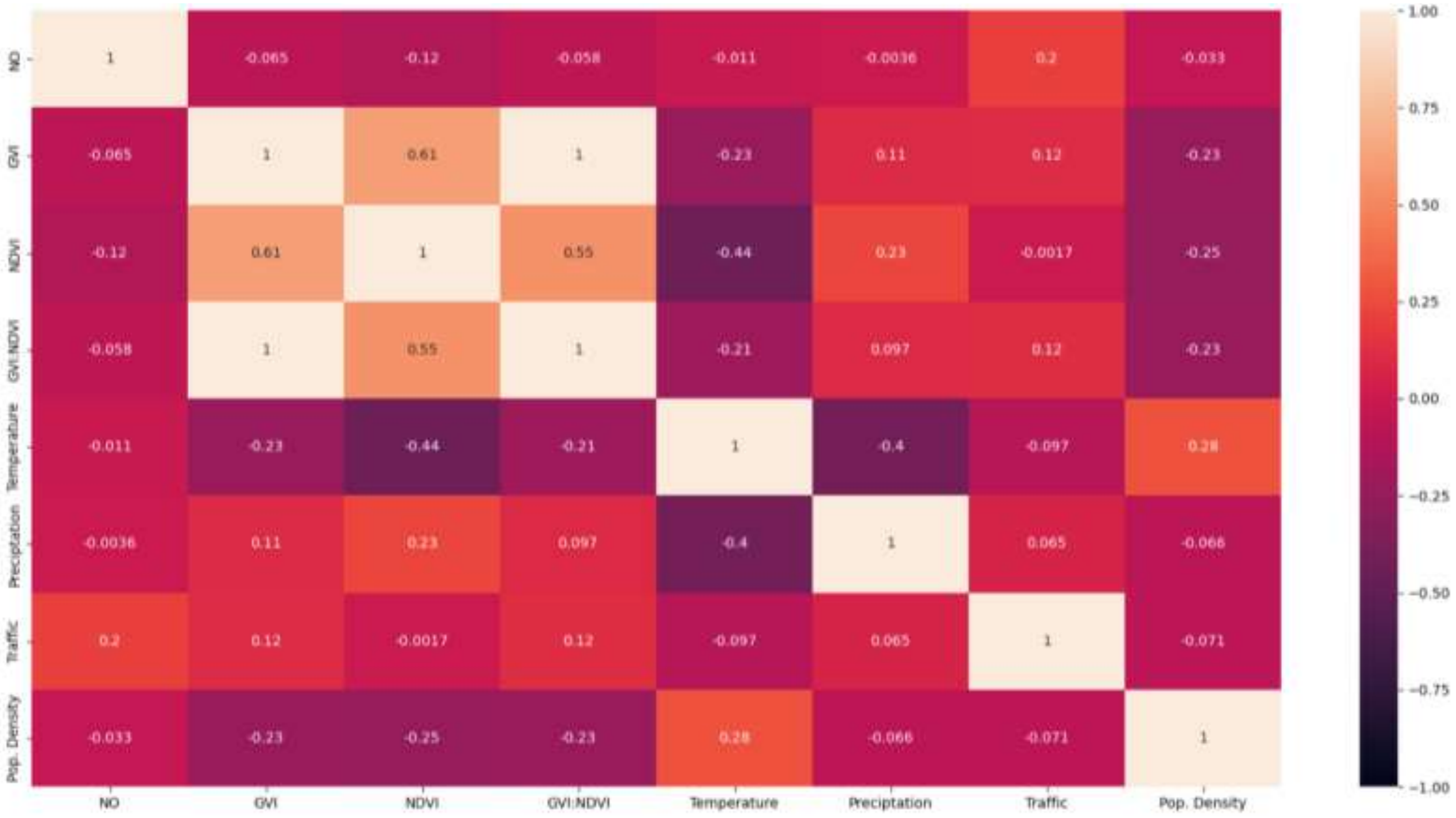

Figure S13. Correlation table for NO, all the urban greenspace metrics (GVI, NDVI, GVI:NDVI), and control variables (temperature, precipitation, traffic and population density), for all point locations (n=24,694).

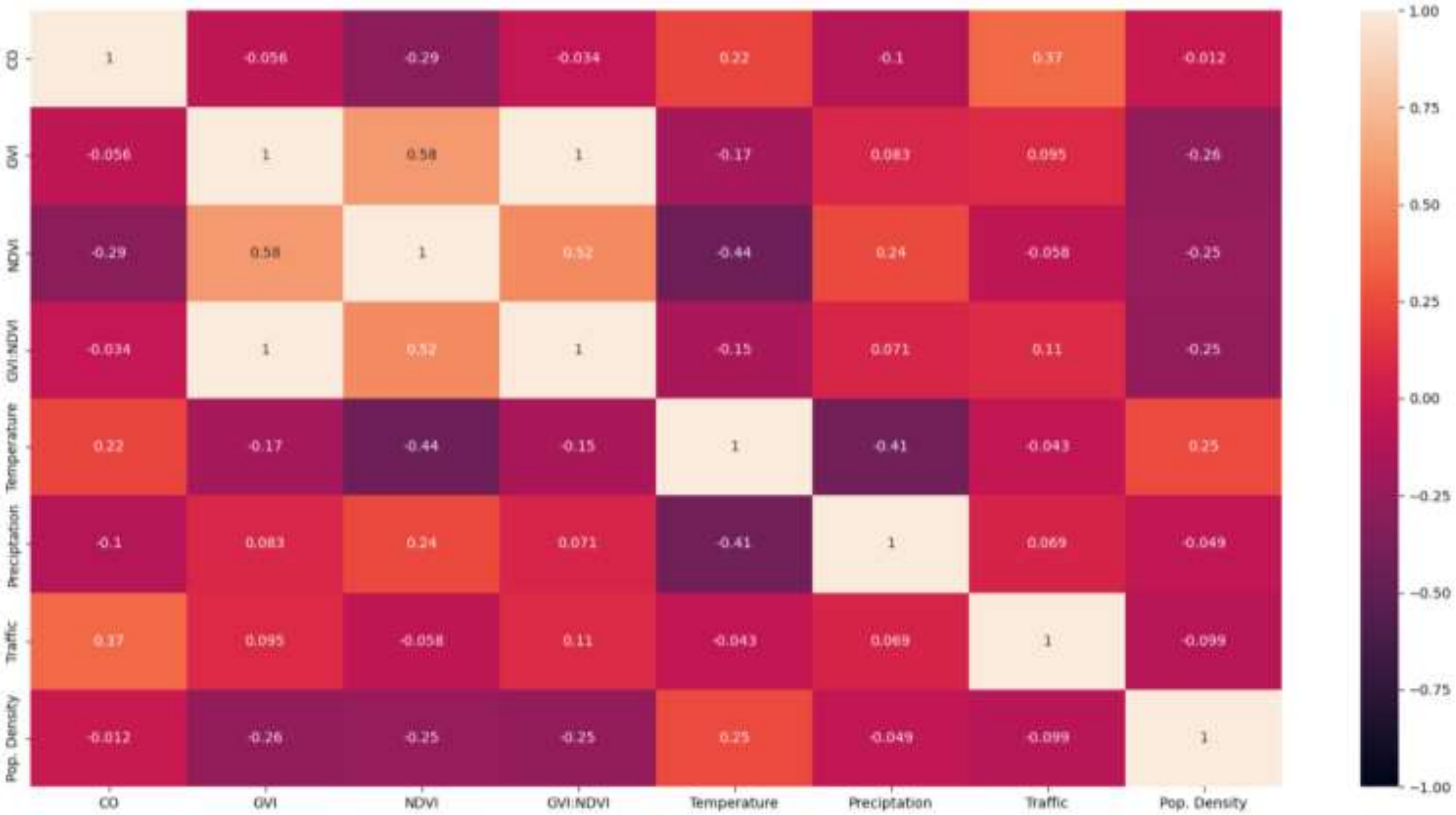

Figure S14. Correlation table for CO, all the urban greenspace metrics (GVI, NDVI, GVI:NDVI), and control variables (temperature, precipitation, traffic and population density), for all point locations (n=24,694).

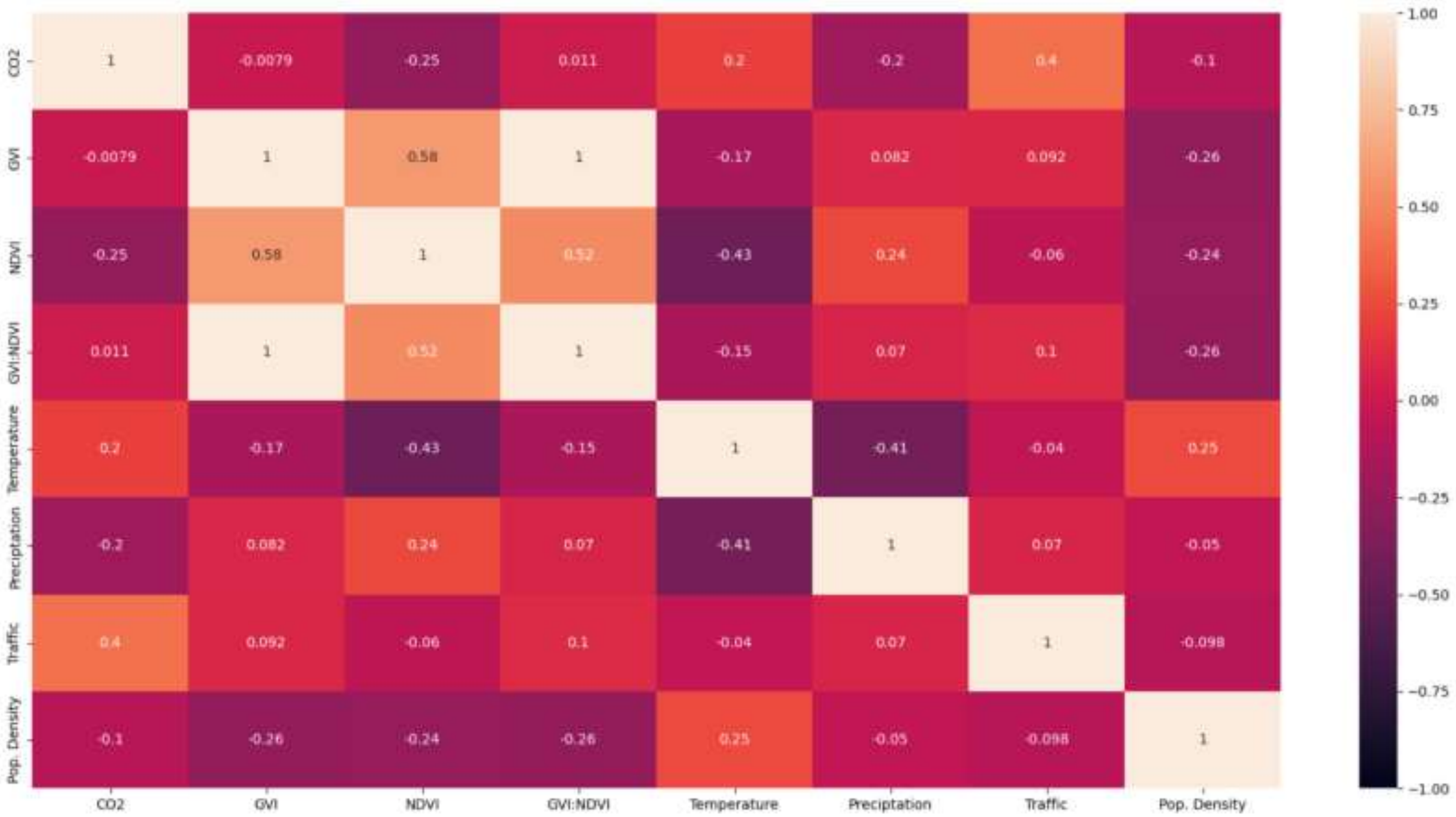

Figure S15. Correlation table for CO_2, all the urban greenspace metrics (GVI, NDVI, GVI:NDVI), and control variables (temperature, precipitation, traffic and population density), for all point locations (n=24,694).

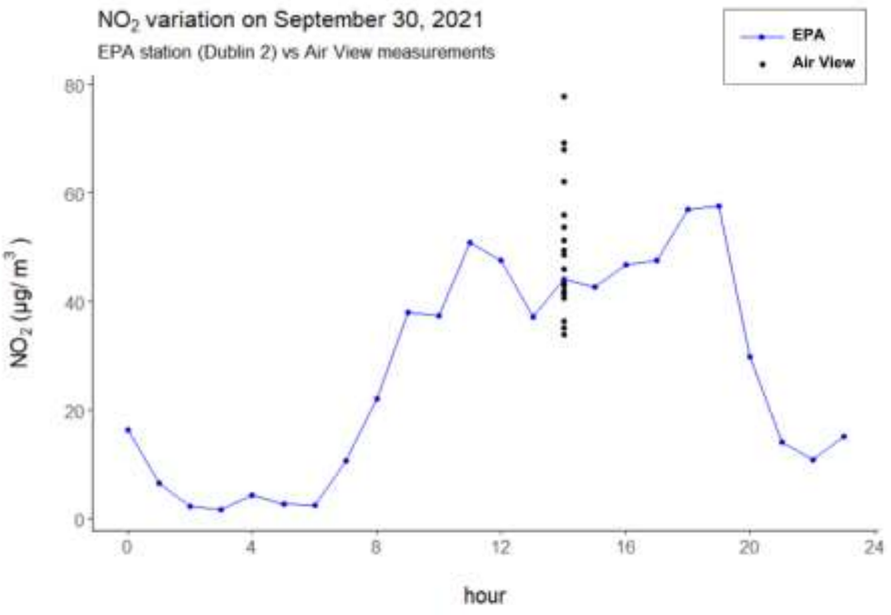

Figure S16. NO_2 measurements for the EPA Station Dublin 2 and Google Air View measurements at the same location for September 30, 2021. EPA measurements are hourly-averaged and available for the whole day, while Air View measurements were only taken between 14:00h and 15:00h. The graph allows the visual inspection of the spatial variation of NO_2 since different concentrations were recorded for each hour.

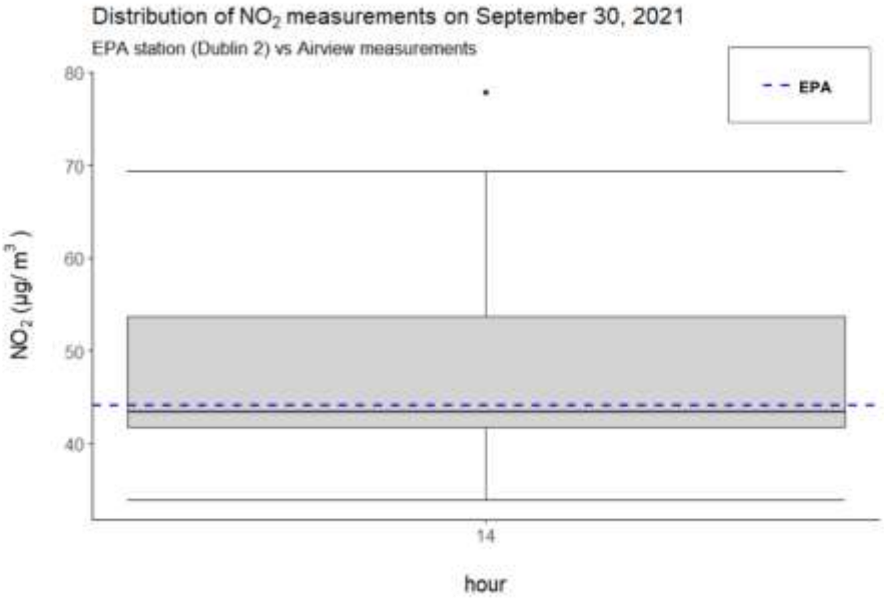

Figure S17. Distribution of NO_2 Google Air View measurements vs. hour-average concentrations recorded by the EPA Station Dublin 2. The boxplot demonstrates the distribution of NO_2 values recorded by Google Air View between 14:00h and 15:00h for September 30, 2021. The dotted blue line represents the NO_2 concentrations recorded by EPA Station Dublin 2 during the same time period.

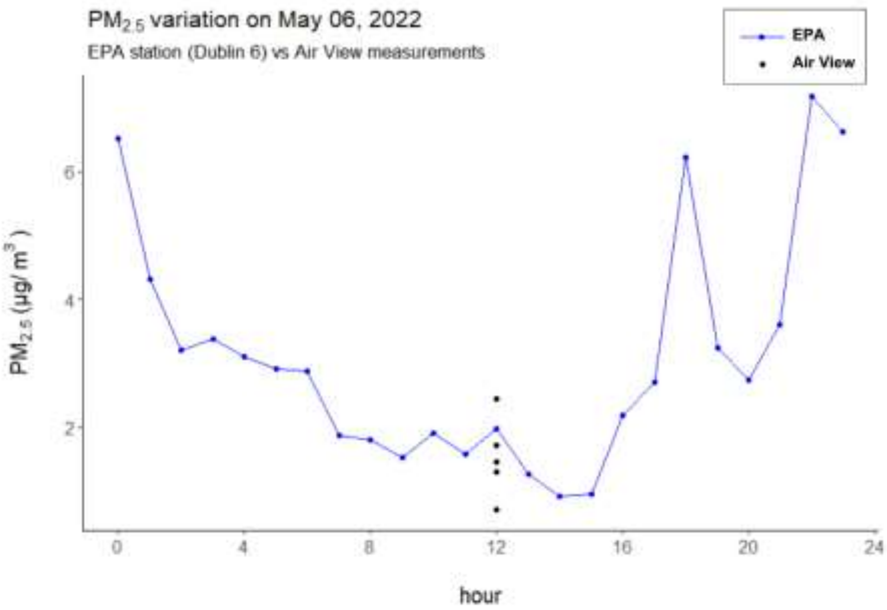

Figure S18. $PM_{2.5}$ measurements for the EPA Station Dublin 6 and Google Air View measurements at the same location for May 06, 2022. EPA measurements are hourly-averaged and available for the whole day, while Google Air View measurements were only taken between 12:00h and 13:00h. The graph allows the visual inspection of the spatial variation of $PM_{2.5}$ since different concentrations were recorded for each hour.

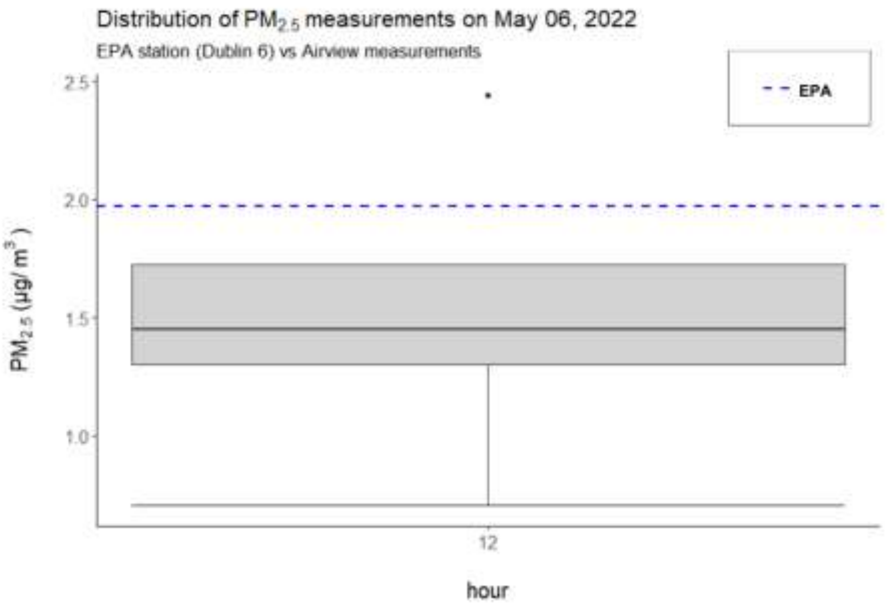

Figure S19. Distribution of $PM_{2.5}$ Google Air View measurements vs. hour-average concentrations recorded by the EPA Station Dublin 6. The boxplot demonstrates the distribution of $PM_{2.5}$ values recorded by Google Air View between 12:00h and 13:00h for May 06, 2022. The dotted blue line represents the $PM_{2.5}$ concentrations recorded by EPA Station Dublin 6 during the same time period.

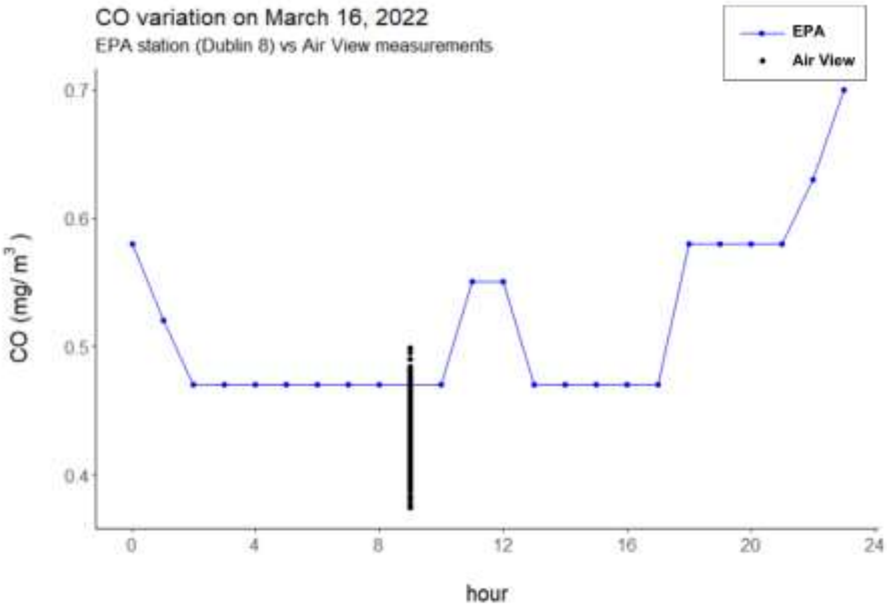

Figure S20. CO measurements for the EPA Station Dublin 8 and Google Air View measurements at the same location for March 16, 2022. EPA measurements are hourly-averaged and available for the whole day, while Google Air View measurements were only taken between 09:00h and 10:00h. The graph allows the visual inspection of the spatial variation of CO since different concentrations were recorded during the day.

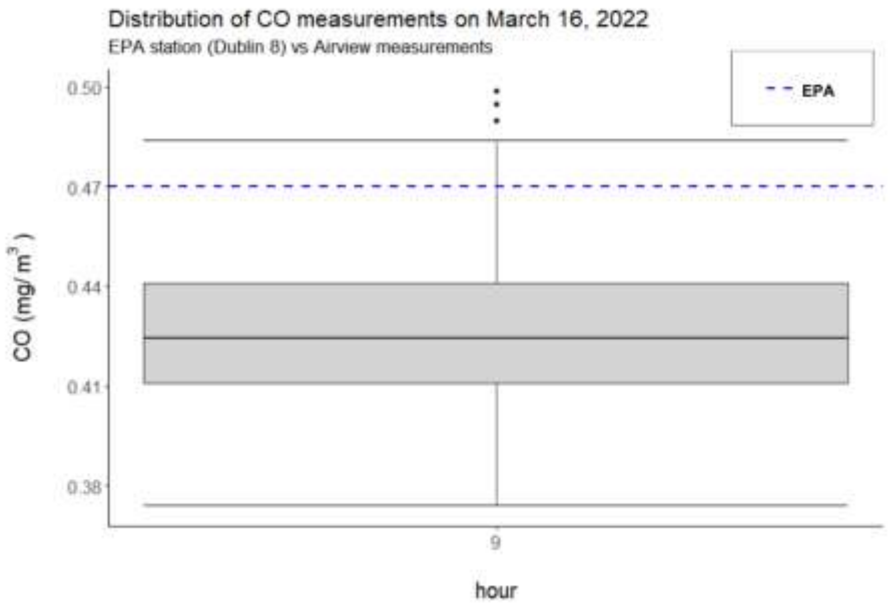

Figure S21. Distribution of CO Google Air View measurements vs. hour-average concentrations recorded by the EPA Station Dublin 8. The boxplot demonstrates the distribution of CO values recorded by Google Air View between 09:00h and 10:00h for March 16, 2022. The dotted blue line represents the CO concentrations recorded by EPA Station Dublin 8 during the same time period.

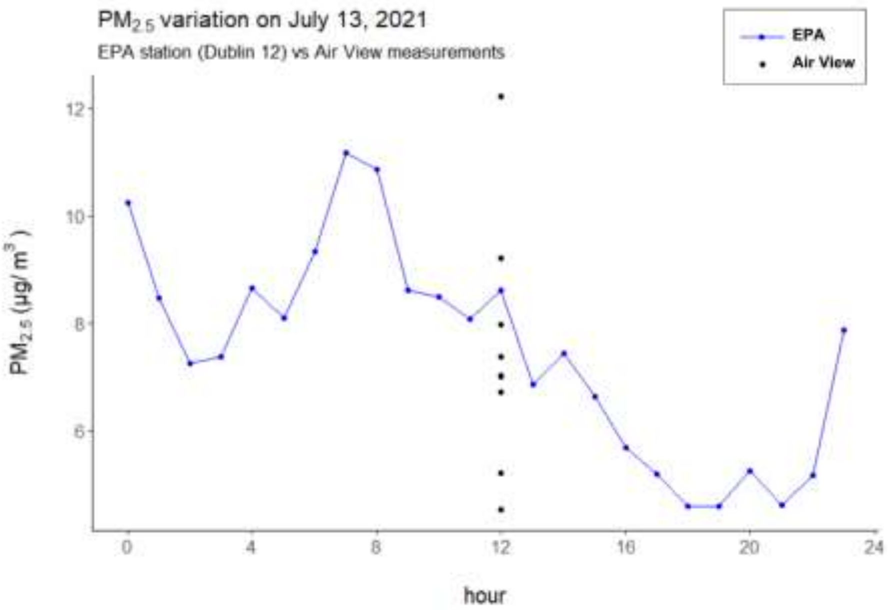

Figure S22. $PM_{2.5}$ measurements for the EPA Station Dublin 12 and Google Air View measurements at the same location for July 13, 2021. EPA measurements are hourly-averaged and available for the whole day, while Google Air View measurements were only taken between 12:00h and 13:00h. The graph allows the visual inspection of the spatial variation of $PM_{2.5}$ since different concentrations were recorded for each hour.

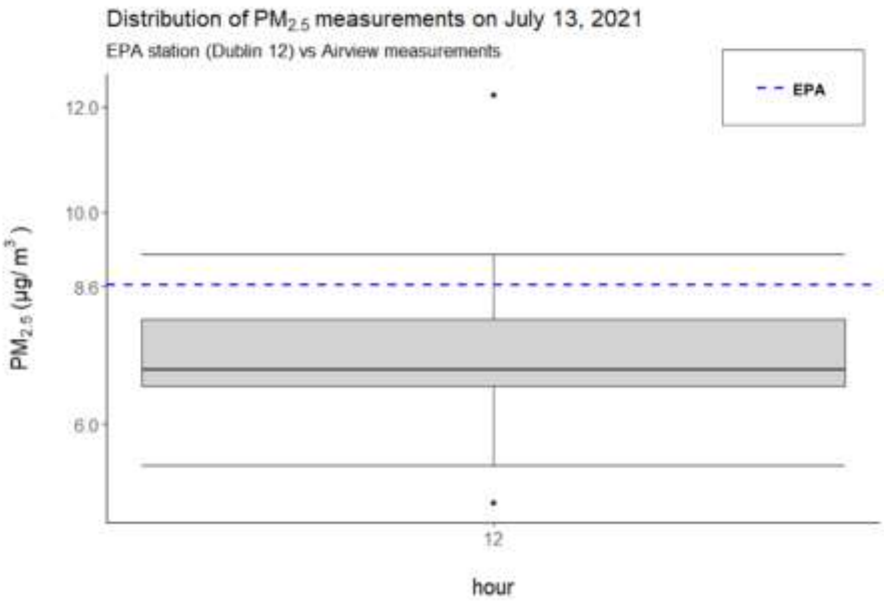

Figure S23. Distribution of $PM_{2.5}$ Google Air View measurements vs. hour-average concentrations recorded by the EPA Station Dublin 12. The boxplot demonstrates the distribution of $PM_{2.5}$ values recorded by Google Air View between 12:00h and 13:00h for July 13, 2021. The dotted blue line represents the $PM_{2.5}$ concentration recorded by EPA Station Dublin 6 during the same time period.

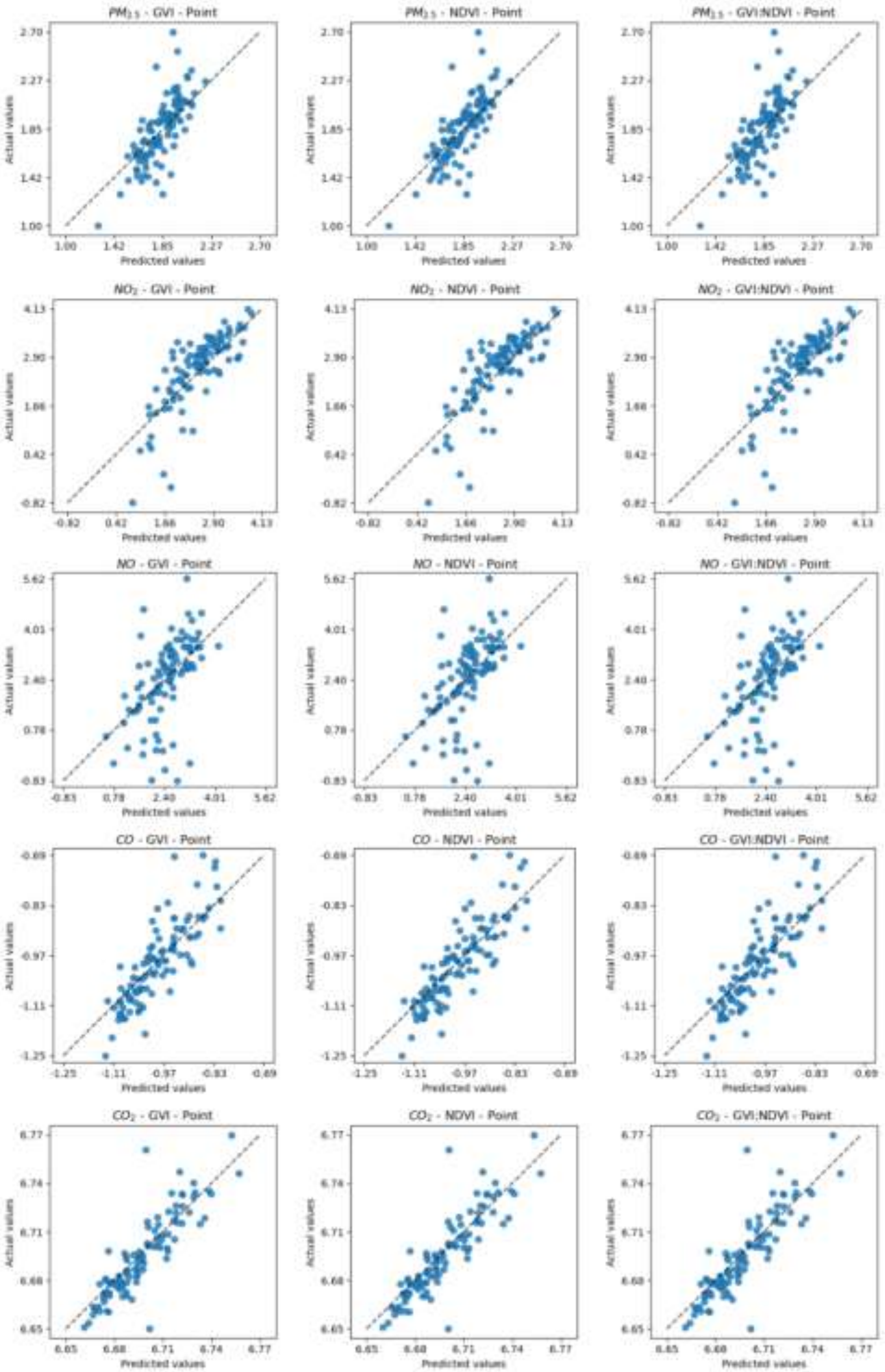

Figure S24. Scatterplots of actual values vs. predicted values for point locations (n=24,694). Spatial lag regression models were used to analyse associations between urban greenspace (GVI, NDVI, GVI:NDVI) and air pollution ($PM_{2.5}$, NO_2, NO, CO and CO_2) levels. All models were adjusted for traffic, precipitation, temperature, and population density. Graphs were plotted with a random sample of 150 points.

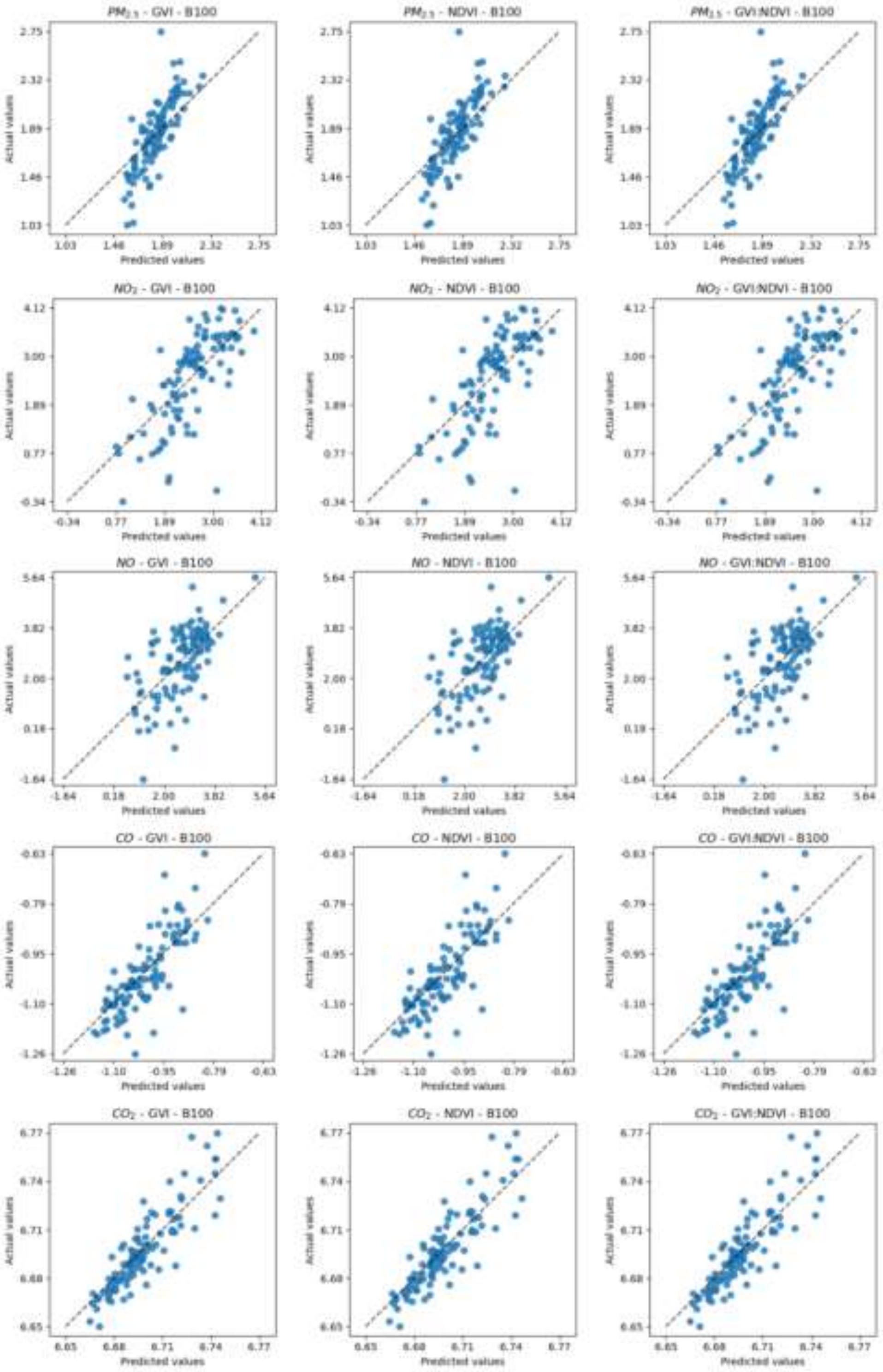

Figure S25. Scatterplots for actual values vs. predicted values for 100m buffer zones (n=24,694). Spatial lag regression models were used to analyse associations between urban greenspace (GVI, NDVI, GVI:NDVI) and air pollution ($PM_{2.5}$, NO_2, NO, CO and CO_2) levels. All models were adjusted for traffic, precipitation, temperature, and population density. Graphs were plotted with a random sample of 150 points.

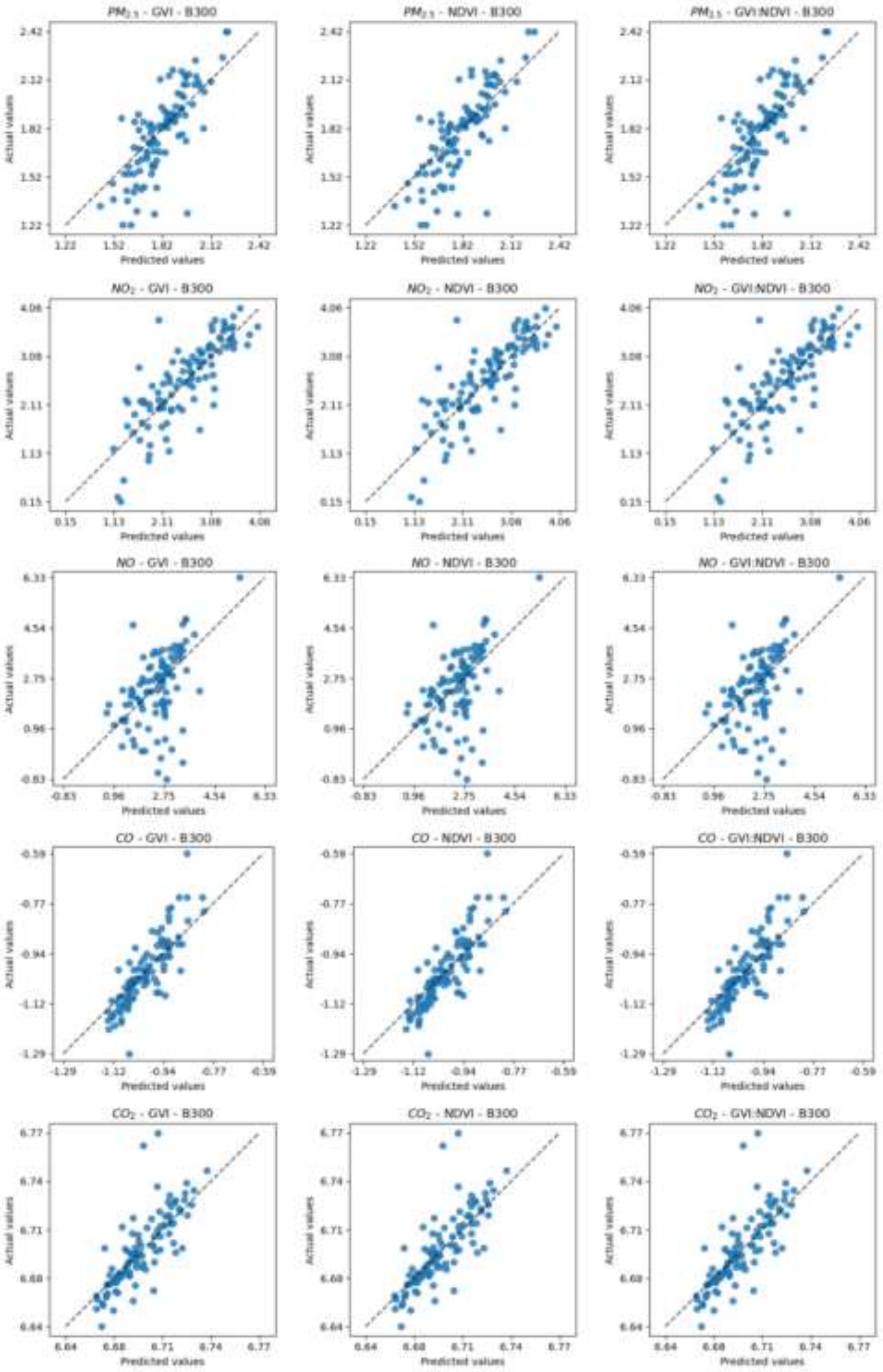

Figure S26. Scatterplots for actual values vs. predicted values for the 300m buffer zones (n=24,694). Spatial lag regression models were used to analyse associations between urban greenspace (GVI, NDVI, GVI:NDVI) and air pollution ($PM_{2.5}$, NO_2, NO, CO and CO_2) levels. All models were adjusted for traffic, precipitation, temperature, and population density. Graphs were plotted with a random sample of 150 points.

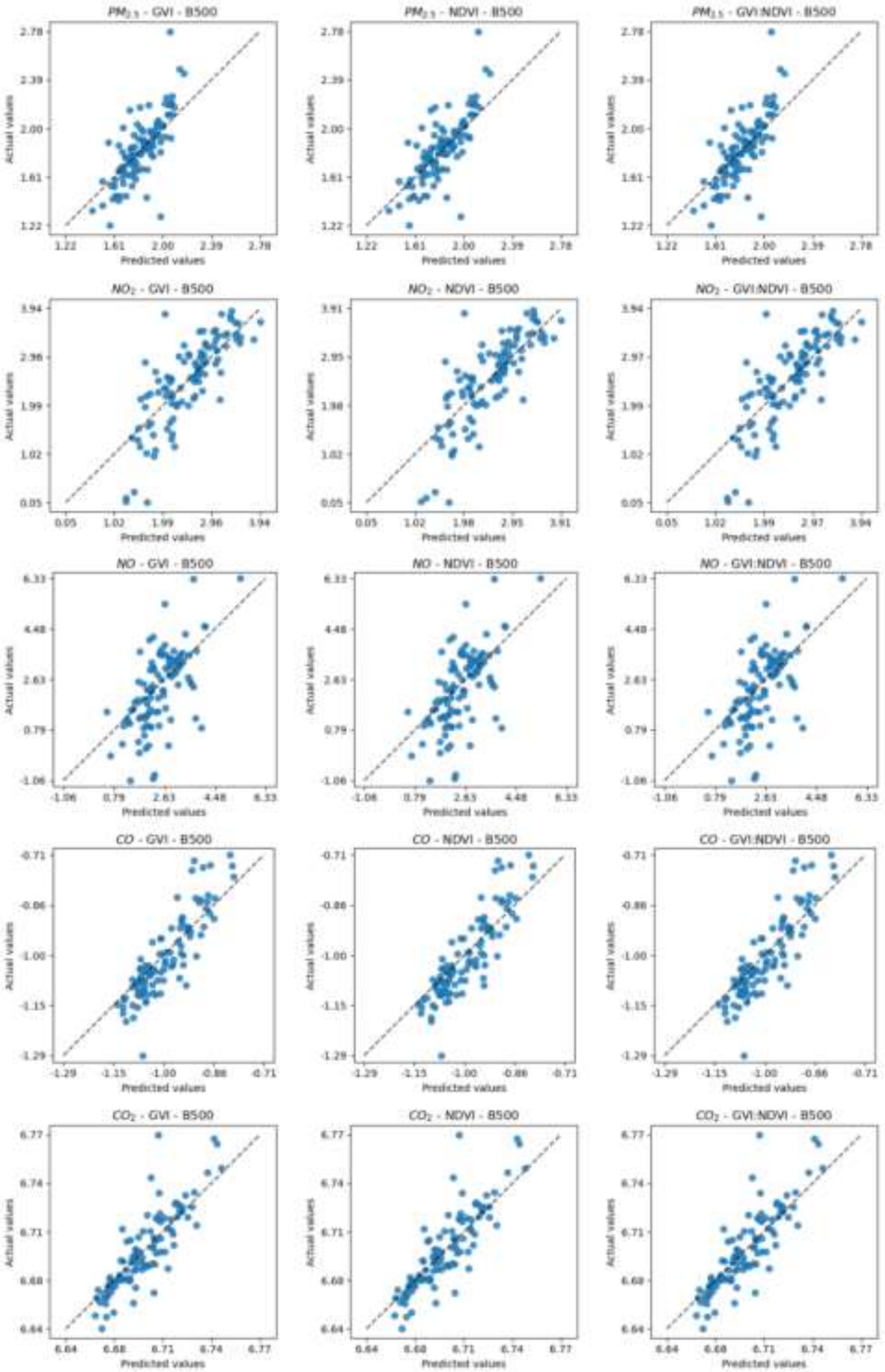

Figure S27. Scatterplots for actual values vs. predicted values for the 500m buffer zones (n=24,694). Spatial lag regression models were used to analyse associations between urban greenspace (GVI, NDVI, GVI:NDVI) and air pollution ($PM_{2.5}$, NO_2, NO, CO and CO_2) levels. All models were adjusted for traffic, precipitation, temperature, and population density. Graphs were plotted with a random sample of 150 points.

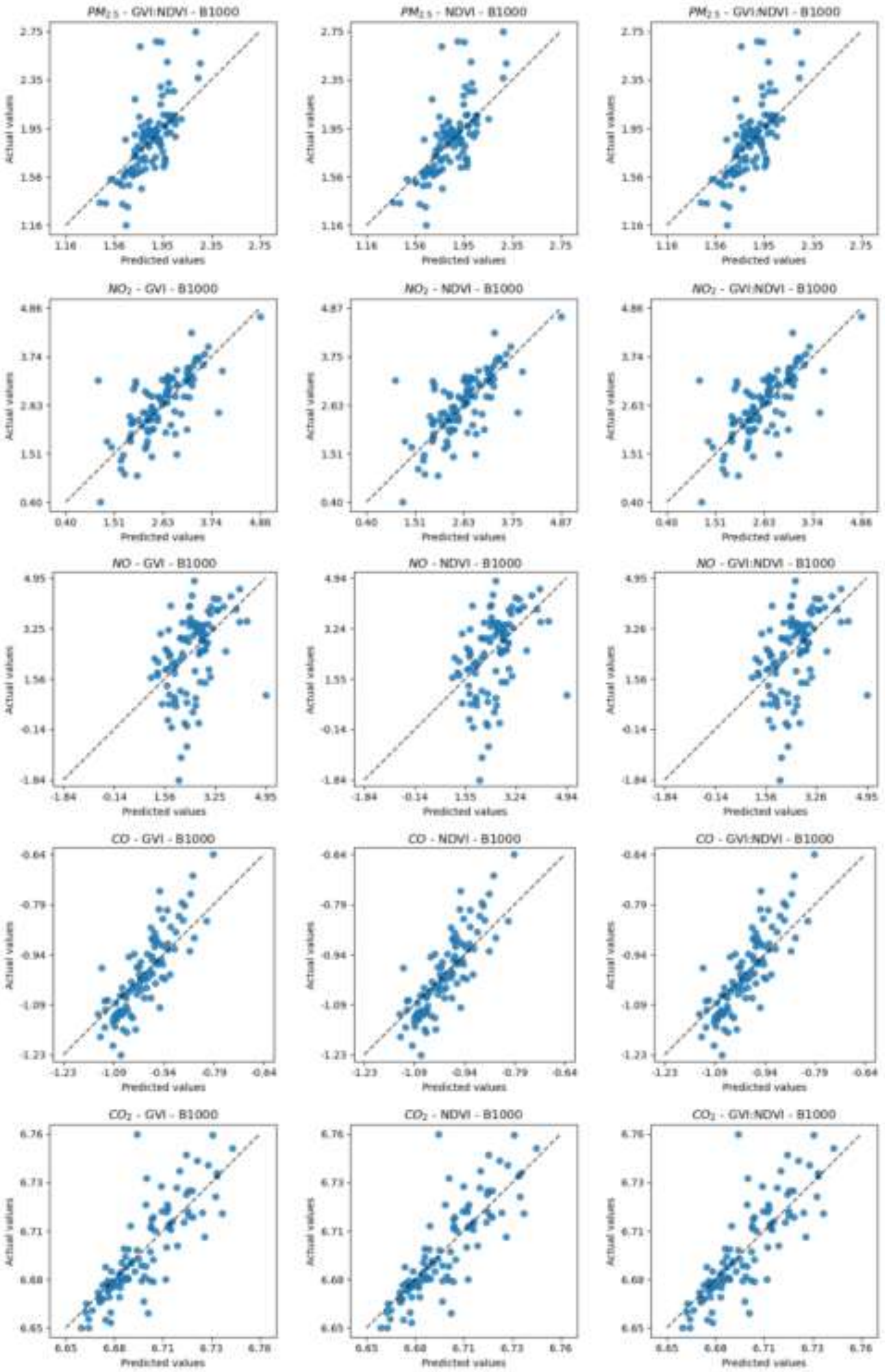

Figure S28. Scatterplots for actual values vs. predicted values for the 1000m buffer zones (n=24,694). Spatial lag regression models were used to analyse associations between urban greenspace (GVI, NDVI, GVI:NDVI) and air pollution ($PM_{2.5}$, NO_2, NO, CO and CO_2) levels. All models were adjusted for traffic, precipitation, temperature, and population density. Graphs were plotted with a random sample of 150 points.

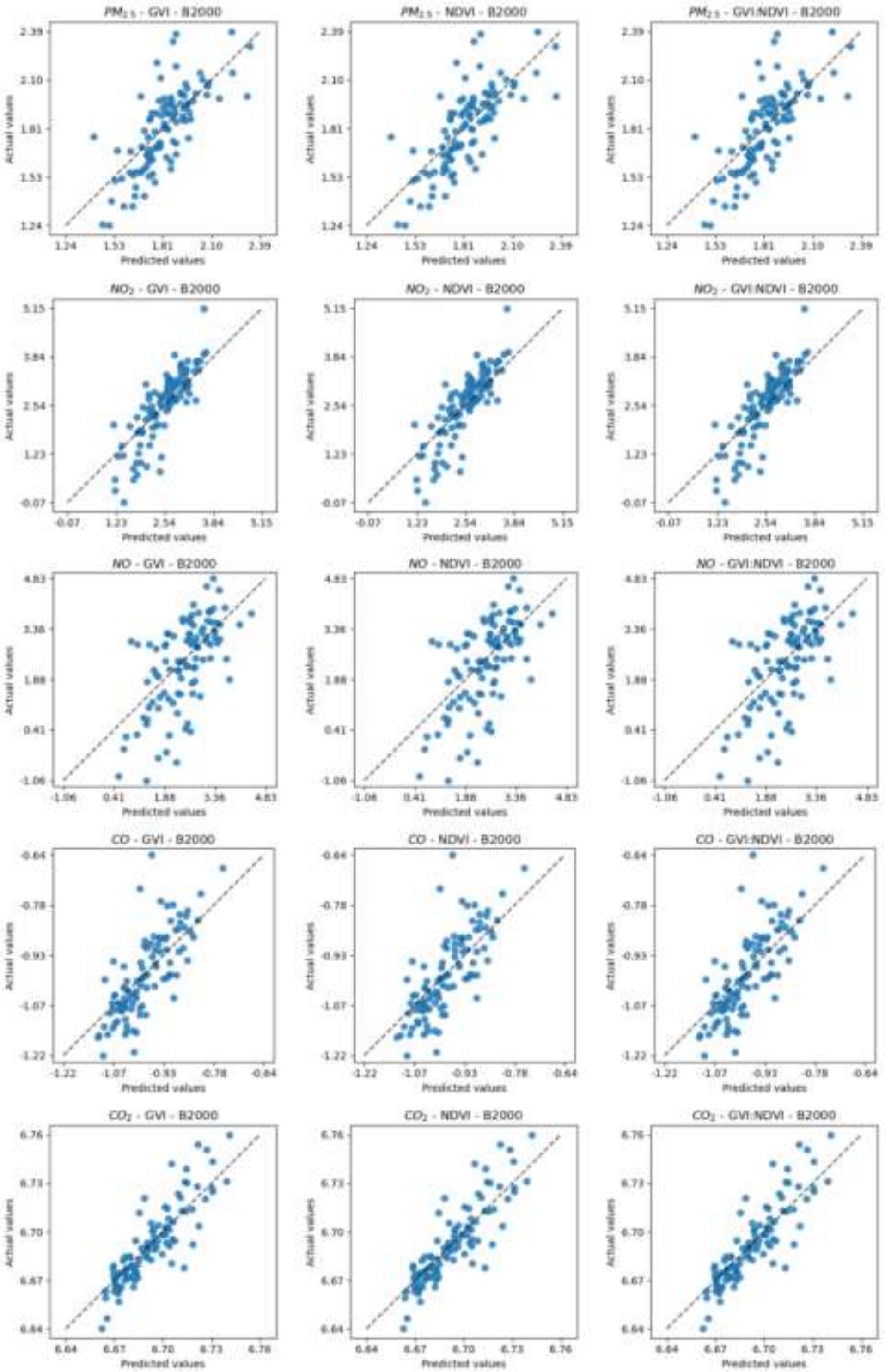

Figure S29. Scatterplots for actual values vs. predicted values for the 200m buffer zones (n=24,694). Spatial lag regression models were used to analyse associations between urban greenspace (GVI, NDVI, GVI:NDVI) and air pollution ($PM_{2.5}$, NO_2, NO, CO and CO_2) levels. All models were adjusted for traffic, precipitation, temperature, and population density. Graphs were plotted with a random sample of 150 points.
